# Supplementary material for: Quantum speedup for nonreversible Markov chains
Source: Nat Commun. 2025 Nov 28;16:10732. doi: 10.1038/s41467-025-65761-5 (PMC12663442; doi:10.1038/s41467-025-65761-5)
Supplement: Supplementary file 1 — Supplementary Information [file 41467_2025_65761_MOESM1_ESM.pdf]

Supplementary information for: Quantum Speedup for Nonreversible Markov Chains

Baptiste Claudon,[1,2,3,*] Jean-Philip Piquemal,[1,3,†] and Pierre Monmarché[2,3,4,‡]

[1] *Qubit Pharmaceuticals, Advanced Research Department, 75014 Paris, France*
[2] *Sorbonne Université, LJLL, UMR 7198 CNRS, 75005 Paris, France*
[3] *Sorbonne Université, LCT, UMR 7616 CNRS, 75005 Paris, France*
[4] *Institut Universitaire de France, 75005 Paris, France*

(Dated: October 22, 2025)

The present document provides a mathematical study of the curved and flat discriminants as well as the polynomials that are applied to their singular values or eigenvalues. Section I introduces Markov chain definitions and basic results. Section II introduces the curved discriminant and details how to polynomially transform its singular values to prepare a reflection through the stationary distribution. Section III generalizes the previous construction to reflect through the leading eigenvector of a symmetric matrix when the corresponding eigenvalue is close to 1. Section IV shows that this regime is precisely the one of interest to reflect through the most reversible distribution of a Markov chain. Section V contains analytic and numerical examples to illustrate the results from the previous sections.

CONTENTS

I. Definitions and notations 2

II. Reversible methods applied to nonreversible problems 4
A. Quantum singular value transformation of the discriminant matrix 5
B. Scaling factors for the fast-forwarding polynomials 6
C. Complementary polynomial 7

III. Preparing the dominant eigenvector of a symmetric matrix 7

IV. Properties of the flat discriminant 9
A. Generic properties 9
B. ϵ-mixed kernels 12
C. Relation between the spectra of the kernel and its discriminant 13
D. Relation between the spectra of the additive reversibilization of the kernel and its discriminant 14
E. Random walks on finite groups 15
F. Flat discriminant and quasi-stationary distributions 16
G. Geometric reversibilization 18

V. Examples 19
A. Exponential speedup for the winning streak Markov chain 19
B. Random walk on a group 19
C. Markov chain on a graph with bottleneck 20
D. Biased random walk and its geometric reversibilization 20
E. Nonreversible walk on the circle 21
1. Intuition 21
2. Second largest eigenvalue of the discriminant 21
3. Scaling of $\langle \pi | D_j | \pi \rangle$ 22
4. Geometric reversibilization 26
5. Example of quantum circuit 26
F. Kinetic walks for sampling 26
G. Out-of-equilibrium kinetic walk 29

* baptiste.claudon@qubit-pharmaceuticals.com
† jean-philip.piquemal@sorbonne-universite.fr
‡ pierre.monmarche@sorbonne-universite.fr

References 29

A. Quantum singular value transformations
1. Projected unitary encoding 30
2. Qubitization 30
3. Hermitianization 31
4. Generalized quantum signal processing 31
5. Generalized quantum eigenvalue transformation 32
6. Generalized quantum singular value transformation 33

B. Amplitude amplification 34

C. Distances between probability measures 34

D. Perron-Frobenius Theorem 34

E. Bauer-Fike Theorem 35

F. Doeblin condition 35

I. DEFINITIONS AND NOTATIONS

The definitions and results given in this Section are standard. Let us start by defining Markov chains.

Definition 1. Let $\mathbb{S}$ be a finite set of size $n \in \mathbb{N}$. A Markov kernel is a function $P : \mathbb{S}^2 \to [0,1]$ such that:

$$\forall x \in \mathbb{S} : \sum_{y \in \mathbb{S}} P(x,y) = 1. \tag{1}$$

Throughout these notes, we will only consider Markov chains on finite state spaces with a single non-degenerate eigenvalue on the unit circle. Moreover, we will assume that the Markov chains are irreducible in the sense of the following Definition 2.

Definition 2. Let P be a Markov kernel on state space $\mathbb{S}$. It is said to be irreducible if and only if:

$$\forall x, y \in \mathbb{S} : \exists t \in \mathbb{N} : P^t(x,y) > 0. \tag{2}$$

The following propositions ensure that the Markov kernels under study are such that P^t converges as $t \to \infty$ to a strictly positive probability distribution on $\mathbb{S}$.

Proposition 1. Let P be a Markov chain with a single non-degenerate eigenvalue on the unit circle. Then, there exists a function $\pi : \mathbb{S} \to [0,1]$ such that:

$$\forall x, y \in \mathbb{S} : \lim_{t \to \infty} P^t(x,y) = \pi(y). \tag{3}$$

In the following proof, $\|\cdot\|_p$ denotes the usual l^p norm defined for every function $f : \mathbb{S} \to \mathbb{C}$ by:

$$\|f\|_p = \left(\sum_{x \in \mathbb{S}} |f(x)|^p \right)^{1/p} \tag{4}$$

for $1 \leq p < \infty$ and $\|f\|_p = \max_{x \in \mathbb{S}} |f(x)|$ if $p = \infty$. The norm $\|\cdot\|$ on complex matrices of size $n \times n$ is the spectral norm.

Proof. Since P contracts the $\|\cdot\|_\infty$ norm, the spectrum of P must lie inside the unit disk. Since the lines of P all sum to 1, the uniform vector is a right eigenvector of P with eigenvalue 1. Let π be a left eigenvector with eigenvalue 1, normalized such that $\|\pi\|_1 = 1$. Let Π be the matrix with π on each its lines: $\Pi(x, y) = \pi(y)$ for all $x, y \in \mathbb{S}$. First, let us show that Π is a Markov kernel (up to multiplying it by a phase). Write π as the limit of a convergent subsequence of the sequence of measures:

$$\begin{cases} \nu_0 = \mu, \\ \nu_{n+1} = \nu_n P, \forall n \geq 0 \end{cases} \tag{5}$$

for any measure μ on $\mathbb{S}$ (such subsequence exists according to the Bolzano-Weierstrass Theorem). It follows that π can be chosen to be a positive vector. Let us now prove that $\sigma(P - \Pi) \subset \sigma(P) \cup \{0\} \backslash \{1\}$. Let f be an eigenvector of $P - \Pi$ with eigenvalue λ, then:

$$\pi(P - \Pi)f = \lambda \pi f \implies 0 = \lambda \pi f. \tag{6}$$

In words, either $\lambda = 0$ or $\pi f = 0$ and $\lambda \in \sigma(P)\backslash\{1\}$. Thus, $\sigma(P - \Pi) \subset \sigma(P) \cup \{0\}\backslash\{1\}$. In particular, the spectral radius ρ of $P - \Pi$ is strictly less than 1: $0 \leq \rho < 1$. However, $\rho = \lim_{t\to\infty} \|(P - \Pi)^t\|^{1/t}$ by Gelfand's formula. As a consequence, $\|P^t - \Pi\| = \|(P - \Pi)^t\| \to 0$. We have proved that:

$$\forall x, y \in \mathbb{S} : \lim_{t\to\infty} P^t(x, y) = \pi(y). \tag{7}$$

□

The above proposition suggests the following definition.

Definition 3. Let P be a Markov kernel on a finite state space $\mathbb{S}$. π, defined as in Proposition 1, is called the stationary distribution of P. Moreover, Π is another Markov kernel on $\mathbb{S}$ such that $\Pi(x, y) = \pi(y)$ for all $x, y \in \mathbb{S}$.

In this study, we are interested in chains such that Π is strictly positive element-wise. This property holds for ergodic chains, defined as follows.

Definition 4. Let P be a Markov chain on a finite state space $\mathbb{S}$. P is said to be ergodic if it is irreducible and has a single non-degenerate eigenvalue on the unit circle.

The following proposition ensures strict positivity of the stationary distribution of an ergodic Markov chain.

Proposition 2. Let P be an ergodic Markov chain, then its stationary distribution π takes values in $]0, 1[$.

Proof. Let $x \in \mathbb{S}$ and $t \in \mathbb{N}$. Recall from the proof of Proposition 1 that π must be a left eigenvector of P with eigenvalue 1:

$$\pi(x) = \sum_{y\in\mathbb{S}} \pi(y)P^t(y, x). \tag{8}$$

Since an eigenvector must be non-zero by definition, there exists $z \in \mathbb{S}$ such that $\pi(z) > 0$. Since P is irreducible, there exists $t \in \mathbb{S}$ such that $P^t(z, x) > 0$. As a consequence, $\pi(x) \geq \pi(z)P^t(z, x) > 0$. □

If a kernel admits a strictly positive left eigenvector, it is possible to define a scalar product induced by this eigenvector.

Definition 5. Define the adjoint $P^\star$ of P as its adjoint according to the scalar product induced by π. More precisely, for $f, g : \mathbb{S} \to \mathbb{C}$, define

$$\langle f, g\rangle_\pi = \sum_{x\in\mathbb{S}} \pi(x)\overline{f(x)}g(x). \tag{9}$$

Then $P^\star$ is the unique operator such that:

$$\forall f, g : \mathbb{S} \to \mathbb{C} : \langle f, Pg\rangle_\pi = \langle P^\star f, g\rangle_\pi\,. \tag{10}$$

For any distribution μ on $\mathbb{S}$, we will denote its corresponding coherent state by $|\mu\rangle$ with $\langle x|\mu\rangle = \sqrt{\mu(x)}$ for each $x \in \mathbb{S}$. In particular, $|\pi\rangle = \sum_{x\in\mathbb{S}} \sqrt{\pi(x)}\,|x\rangle$ is the coherent state corresponding to the stationary distribution. We may refer to both μ and $|\mu\rangle$ as the distribution. The presence or not of the $|\cdot\rangle$ notation will make the actual subject clear. $\langle\cdot,\cdot\rangle$ without the π subscripts corresponds to a standard hermitian product:

$$\forall f, g : \mathbb{S} \to \mathbb{C} : \langle f, g\rangle = \sum_{x\in\mathbb{S}} \overline{f(x)} g(x). \tag{11}$$

Note that when an operator H is symmetric, $H(x,y) = \overline{H(y,x)}$ for each $x, y \in \mathbb{S}$, we will adopt the Dirac notation:

$$\langle f|H|g\rangle = \langle f, Hg\rangle = \langle Hf, g\rangle \tag{12}$$

for each $f, g : \mathbb{S} \to \mathbb{C}$. Let us now introduce two ways to measure the distance between probability distributions, starting with the most common total variation distance.

Definition 6. Let μ and ν be probability distributions on $\mathbb{S}$. The total variation distance between μ and ν is defined to be:

$$d_{TV}(\mu,\nu) = \frac{1}{2}\|\mu - \nu\|_1. \tag{13}$$

The Hellinger distance between two distributions is related to their overlap as follows.

Definition 7. Let μ and ν be probability distributions on $\mathbb{S}$. The Hellinger distance between μ and ν is defined to be:

$$d_H(\mu,\nu) = \sqrt{1 - \langle\mu|\nu\rangle}. \tag{14}$$

Proposition 1 implies that for each probability distribution μ on $\mathbb{S}$, $d_{TV}(\mu P^t, \pi) \to 0$ as $t \to \infty$. In particular, the following definition is well-posed.

Definition 8. Let $0 < \epsilon < 1$. Denote the ϵ-mixing time of a Markov chain P by:

$$\tau(\epsilon) = \min_{t\in\mathbb{N}} \left\{ t : \max_{x\in\mathbb{S}} d_{TV}\left(P^t(x,\cdot), \pi\right) \le \epsilon \right\}. \tag{15}$$

The following quantities will also appear in the analysis.

Definition 9. The Poincaré constant $\gamma(P)$ is defined by:

$$\gamma(P) = \min_{f:\pi f=0} \langle f, (I-P)f\rangle_\pi. \tag{16}$$

Definition 10. Define the absolute spectral gap δ of a Markov kernel P by:

$$\delta = 1 - \max_{\lambda\in\sigma(P)\setminus\{1\}} |\lambda|. \tag{17}$$

From now on, we only consider ergodic Markov kernels. We might also locally (but explicitly and without loss of generality) assume that a kernel is lazy, in the following sense.

Definition 11. Let P be a Markov kernel with state space $\mathbb{S}$. It is said to be lazy if:

$$\forall x \in \mathbb{S} : P(x,x) \ge 1/2. \tag{18}$$

II. REVERSIBLE METHODS APPLIED TO NONREVERSIBLE PROBLEMS

This section shows how to use reflections through the laws of P and those through the laws of its adjoint $P^\star$ to construct a projected unitary encoding of the curved discriminant, a matrix that is similar to the kernel P (see Appendix A for quantum signal processing definitions and results). Quantum singular value transformation algorithms then allow to construct a reflection operator through the stationary distribution. The complexity of the procedure depends on the singular value gap and on a so-called scaling factor introducing a probability of failure. This section compares the resulting complexity of generating this reflection with the mixing time of the chain. In order to do so, it uses modern results on nonreversible Markov chains and shows that the applied singular value transformation has full probability of success.

A. Quantum singular value transformation of the discriminant matrix

Let us start by properly defining discriminants [1].

Definition 12. Let (P, Q) be Markov kernels on a finite state space $\mathbb{S}$. The associated discriminant D is defined by its matrix elements:

$$\forall x, y \in \mathbb{S} : D(x, y) = \sqrt{P(x, y)Q(y, x)}. \tag{19}$$

The curved discriminant of a Markov chain is the discriminant associated with the pair $(P, P^\star)$ of Markov kernels.

Definition 13. Define the discriminant matrix of a Markov kernel P by its matrix elements:

$$\forall x, y \in \mathbb{S} : \mathcal{D}(x, y) = \sqrt{P(x, y)P^\star(y, x)}. \tag{20}$$

The following Proposition 3 is obvious but so useful that it deserves a separate statement.

Proposition 3. $\mathcal{D}$ is similar to P.

Proof. Let $x, y \in \mathbb{S}$. Then,

$$\mathcal{D}(x, y) = \sqrt{\frac{\pi(x)}{\pi(y)}} P(x, y) = \left(MPM^{-1}\right)(x, y), \tag{21}$$

where $M(w, z) = \delta_{w,z}\sqrt{\pi(w)}$ for all $w, z \in \mathbb{S}$. □

There is an obvious generalization of the known quadratic speedup for reversible chains. It is a corollary from the following Proposition 4.

Proposition 4. Let P be a kernel such that $P^\star P = PP^\star$ (normal with respect to $\langle \cdot, \cdot \rangle_\pi$). Then, the second largest singular value of $\mathcal{D}$ is $1 - \delta$, where δ is the absolute spectral gap of P.

Proof. Proposition 3 implies that $\mathcal{D}^\dagger\mathcal{D}$ is similar to $P^\star P$. Since the eigenvalues of $P^\star P$ are precisely the modulus squared of those of P, the result follows. □

The lower bound $\tau(\epsilon) \in \Omega\left(\delta^{-1}\log(1/\epsilon)\right)$ ensures that the preparation of $2\left|\pi\right\rangle\left\langle\pi\right| - 1$ is quadratically smaller than the mixing time for normal kernels. Indeed, there exists a polynomial (given in Corollary 2) of degree $\mathcal{O}\left(\delta^{-1/2}\log(1/\epsilon)\right)$ that separates the singular value 1 of $\mathcal{D}$ from the others [2]. Generally, the singular values of $\mathcal{D}$ are the singular values of P with respect to $l^2(\pi)$. Since there is no simple relation between the mixing time of a nonreversible kernel and its absolute spectral gap, we will make use of the following notions of spectral gap and relaxation time [3].

Definition 14. Define the generator of a Markov chain L by $L = I - P$. Define the spectral gap ι of the chain as the second smallest singular value of L with respect to $l^2(\pi)$. Define the relaxation time of the chain by $\mathcal{T} = 1/\iota$.

The relaxation time can be characterized as the time required for convergence of empirical averages to their π average. Recall the following theorem.

Theorem 1. Let ι be the spectral gap of the Markov chain with transition matrix P. Denote by ι_M the spectral gap of $M = P^\star P$. Then, $\frac{1}{2}\iota_M \leq \iota$. Moreover, if P is lazy $\frac{1}{6}\iota^2 \leq \iota_M$.

Theorem 1 provides an upper bound for the second largest singular value of the curved discriminant.

Corollary 1. If P is lazy, the singular values $\sigma \neq 1$ of $\mathcal{D}$ verify:

$$\sigma \leq \sqrt{1 - \frac{\iota^2}{6}}. \tag{22}$$

Proof. For each singular value $\sigma \neq 1$ of $\mathcal{D}$, there is an eigenvalue λ of $P^\star P$ such that $\sigma = \sqrt{\lambda}$. However, $\lambda \leq 1 - \iota_M$. Therefore:

$$\sigma \leq \sqrt{1 - \iota_M} \leq \sqrt{1 - \frac{\iota^2}{6}} = 1 - \frac{\iota^2}{12} + \mathcal{O}(\iota^4). \tag{23}$$

□

Therefore, the reflection through the stationary distribution can be constructed with $\mathcal{O}(\mathcal{T}\log(1/\epsilon))$ steps. On the other hand, the best known upper bound of the mixing time is $\mathcal{O}\left(\mathcal{T}^2\log\left(\frac{1}{\epsilon\pi_\star}\right)\right)$. Amplitude amplification algorithms can prepare $\left|\pi\right\rangle$ from $\left|u\right\rangle$ with $1/\left\langle\pi|u\right\rangle$ (which is in $\in \mathcal{O}(\sqrt{n})$ if u is the uniform distribution) uses of the reflection and $\left|u\right\rangle$-state preparation unitary [4].

B. Scaling factors for the fast-forwarding polynomials

Scaling factors are defined in Definition 27. Recall that for each polynomial $\upsilon(x) = \sum_{k=0}^{d} a_k T_k(x)$ with complex coefficients defined in the Chebyshev basis, Υ is defined by: $\Upsilon(z) = \sum_{k=0}^{d} a_k z^k$. More formally, define the linear operator $A : \mathbb{C}[x] \to \mathbb{C}[x]$ by its action on the basis of Chebyshev polynomials:

$$\forall k \in \mathbb{N} : A(T_k(x)) = x^k. \tag{24}$$

The previous definition simply becomes $\Upsilon = A\upsilon$. The scaling factor β of υ is defined by:

$$\beta = \frac{\max_{z \in \partial B_1(0)} |\Upsilon(z)|}{\max_{x \in [-1,1]} |\upsilon(x)|}. \tag{25}$$

Proposition 5. Fix $c \geq 1$ and consider the sequence of polynomials $\upsilon_k(x) = T_k(cx)$ for $k \in \mathbb{N}$. Denote by β_k their scaling factors. Then:

$$\forall k \in \mathbb{N} : \beta_k = 1. \tag{26}$$

Proof. Let $z \in \partial B_1(0)$ and for each $k \in \mathbb{N}$, $\Upsilon_k = A\upsilon_k$. Because $T_k(\cos(\theta)) = \frac{e^{ik\theta}+e^{-ik\theta}}{2}$ for every $\theta \in \mathbb{R}$ and $k \in \mathbb{N}$ (and all the coefficients of $\upsilon_k(x)$ in the Chebyshev basis are real):

$$\forall k \in \mathbb{N} : \mathrm{Re}(\Upsilon_k(z)) = \upsilon_k(\mathrm{Re}(z)). \tag{27}$$

As a consequence, $\beta_k \geq 1$ and there only remains to show that $\beta_k \leq 1$. Expand $\upsilon_k(x)$ on the Chebyshev basis as $\upsilon_k(x) = \sum_{j=0}^{k} a_j^{(k)} T_j(x)$. Notice that the $(a_j^k)_{j,k\in\mathbb{Z}}$ coefficients are all real and that $j > k$ implies $a_j^{(n)} = 0$. Similarly, if j and k have different parity, $a_j^{(n)} = 0$. By convention, $a_j^{(k)} = 0$ for any $k \in \mathbb{N}, j < 0$. We will show by induction on $k \in \mathbb{N}$ that $\forall k, j \in \mathbb{N}$: $a_j^{(k)} \geq 0$. By H_k, we will denote the statement: all the coefficients of $\upsilon_k(x)$ in the Chebyshev basis are positive. Start by computing the first coefficients:

$$\begin{cases} a_0^{(0)} = 1 \\ a_0^{(1)} = 0, a_1^{(1)} = c. \end{cases} \tag{28}$$

In particular, H_0 and H_1 hold. Now, notice that:

$$\upsilon_{k+1}(x) = T_{k+1}(cx) = 2cxT_k(cx) - T_{k-1}(cx). \tag{29}$$

Using that for all $j \geq 1 : 2T_1(x)T_j(x) = T_{j+1}(x) + T_{j-1}(x)$, we get that $a_k^{(k)} = c^k \geq 0$ for all $k \in \mathbb{N}$. Let us now assume that $k \geq 1$ and that for all $m \leq k$, H_m holds. Let $0 \leq j \leq k-1$ be of the same parity as $k+1$. Then,

$$a_j^{(k+1)} = c\left(a_{j+1}^{(k)} + a_{j-1}^{(k)}\right) - a_j^{(k-1)}. \tag{30}$$

If $j+1 = k$, $a_j^{(k+1)} = c^{k+1} - c^{k-1} + ca_{j-1}^{(k)} \geq 0$ because H_k is assumed to hold and $c \geq 1$. If $j < k-1$,

$$\begin{aligned} a_j^{(k+1)} &= ca_{j-1}^{(k)} + c\left(ca_j^{(k-1)} + ca_{j+2}^{(k-1)} - a_{j+1}^{(k-2)}\right) - a_j^{(k-1)} \\ &= ca_{j-1}^{(k)} + (c^2-1)a_j^{(k-1)} + c\left(ca_{j+2}^{(k-1)} - a_{j+1}^{(k-2)}\right) \\ &\geq c\left(ca_{j+2}^{(k-1)} - a_{j+1}^{(k-2)}\right), \end{aligned} \tag{31}$$

using again that H_k and H_{k-1} are assumed to hold and that $c \geq 1$. Repeat $m \in \mathbb{N}$ times until $j+1+m = k-m$ (which will occur since j is of the same parity as k by definition) to get:

$$a_j^{(k+1)} \geq c^m\left(ca_{j+1+m}^{(k-m)} - a_{j+m}^{(k-m-1)}\right) = c^{k+1} - c^{k-1} \geq 0. \tag{32}$$

Summarizing, the property H_k holds for every positive integer k. Using the triangle inequality:

$$\forall z \in \partial B_1(0) : |\Upsilon_k(z)| \leq \sum_{j=0}^{k} \left|a_j^{(k)} z^j\right| = \sum_{j=0}^{k} a_j^{(k)} = \sum_{j=0}^{k} a_j^{(k)} T_j(1) = T_k(c) = \max_{x \in [-1,1]} |\upsilon_k(x)|. \tag{33}$$

Indeed, the derivative of $x \mapsto T_k(cx)$ is known to vanish $k-1$ times in the interval $[-1/c, 1/c]$. Being a polynomial of degree $k-1$, it does not vanish outside this interval. $x \mapsto T_k(cx)$ being increasing at $x = 1/c$, the derivative remains positive in $[1/c, 1]$. In particular, $\max_{z \in \partial B_1(0)} |\Upsilon_k(z)| \leq \max_{x \in [-1,1]} |\upsilon_k(x)|$ and $\beta_k \leq 1$. □

The above proposition directly implies that the scaling factor of the polynomial of interest is 1, allowing us to skip the scaling procedure. This polynomial υ_k is defined on Corollary 2 in such that it less than ϵ on the interval $[-1+\delta, 1-\delta]$, for $\delta \in \mathcal{O}(1/k^2)$.

Corollary 2. The polynomial $\upsilon_k(x) = \epsilon T_k(xT_{1/n}(1/\epsilon))$ has scaling factor $\beta_k = 1$ for any $k \geq 1$ and $0 < \epsilon \leq 1$. Moreover, $\max_{x\in[-1,1]} |\upsilon_k(x)| = 1$.

C. Complementary polynomial

An analytic expression for a canonical complementary polynomial of Υ can be found in [5]. Evaluating this expression requires the knowledge of the roots of $z \mapsto 1 - |\Upsilon(z)|^2$. Here, the roots are known to be ± 1. As a consequence, the complementary polynomial of Υ of degree d may be computed with only logarithmic overhead in $\mathcal{O}(d\log(d))$ time.

III. PREPARING THE DOMINANT EIGENVECTOR OF A SYMMETRIC MATRIX

The polynomial to apply to the singular values of the discriminant is exactly 1 at $x = 1$. But we will see in Section IV that another kind of discriminant needs to be used in case the adjoint operator of the kernel we are interested in is not known. This discriminant has a leading eigenvalue that is only close to 1. This section shows that composing the usual polynomial with a step function yields a polynomial that can separate the eigenvalues of this new operator with the same complexity in the limit where the leading eigenvalues are close enough from 1. This regime is precisely the one of interest, as will be shown in later sections.

The following proposition is well-known [2]. Let $\Theta : \mathbb{R} \to \mathbb{R}$ denote the Heaviside function defined by $\Theta(x) = 1$ if $x \geq 0$ and $\Theta(x) = 0$ otherwise.

Proposition 6. Let $a \in]-1, 1[$ and $0 < \Delta, \epsilon < 1$. There exists a polynomial $q_{a,\Delta,\epsilon}$ of degree $m \in \mathcal{O}(\Delta^{-1}\log(1/\epsilon))$ such that $|q_{a,\Delta,\epsilon}| \leq 1$ on $[-1, 1]$ and $\|q_{a,\Delta,\epsilon} - \Theta(\cdot - a)\|_{\infty,[-1,a-\Delta/2]\cup[a+\Delta/2,1]} \leq \epsilon$.

The following Theorem is inspired by [6].

Theorem 2. Let $0 < \epsilon < 1$. Let $(\delta_k)_{k\in\mathbb{N}}$ be the sequence of positive real numbers defined for each $k \in \mathbb{N}$ by:

$$\delta_k = \frac{T_{1/k}(1/\epsilon) - 1}{T_{1/k}(1/\epsilon)}, \tag{34}$$

where $T_y : x \mapsto \cosh\left(y\cosh^{-1}(x)\right)$ for each $y \in]0, 1[$. Let $\mathcal{C}(\epsilon, k)$ be the set of defined parity real polynomials υ having all their roots in $]-1, 1[$ an such that:

- $\upsilon(1) = 1$,
- $\forall x \in [-1+\delta_k, 1-\delta_k]$, $|\upsilon(x)| \leq \epsilon$.

Then, there is no polynomial of degree less than k in $\mathcal{C}(\epsilon, k)$. Moreover, the polynomial $\upsilon_{\epsilon,k}(x) = \epsilon T_k(T_{1/k}(1/\epsilon)x)$ is such that $\max \upsilon_{\epsilon,k}^{-1}(\epsilon) = 1 - \delta_k$ and the only polynomial of degree k in $\mathcal{C}(\epsilon, k)$.

Proof. Let $Q \in \mathcal{C}(\epsilon, k)$. Let $D = Q - \upsilon_{\epsilon,k}$. Let $(y_j)_{j=0}^k$ be the points in $[-1+\delta_k, 1-\delta_k]$ where $|\upsilon_{\epsilon,k}|$ reaches its maximum ϵ. Define $(y_j)_{j=0}^k$ so that the points are in increasing order. Because $Q \in \mathcal{C}(\epsilon, k)$ by hypothesis,

$$(-1)^k D(y_0) \leq 0, (-1)^{k+1} D(y_1) \leq 0, ..., -D(y_{k-1}) \leq 0, D(y_k) \leq 0. \tag{35}$$

As a consequence, D has at least k roots in $[-1+\delta_k, 1-\delta_k]$. Since 1 is also a root of D, either $D = 0$ or D is of degree at least $k+1$. In other words, either $Q = \upsilon_{\epsilon,k}$ or Q is of degree at least $k+1$. □

Lemma 1. Let $(\delta_k)_{k\in\mathbb{N}}$ be defined as in Theorem 2. The following estimate holds as $k \to \infty$:

$$k = \frac{\cosh^{-1}(1/\epsilon)}{\sqrt{2\delta_k}} + \mathcal{O}(1). \tag{36}$$

Proof. Using that $\cosh^{-1}(x) = \ln\left(x + \sqrt{x^2-1}\right)$ for $x \geq 1$:

$$\begin{aligned}
&T_{1/k}(1/\epsilon)(1-\delta_k) = 1 \\
\implies &\frac{1}{k}\cosh^{-1}(1/\epsilon) = \cosh^{-1}(1/(1-\delta_k)) \\
\iff &\frac{1}{k}\cosh^{-1}(1/\epsilon) = \ln\left(1 + \sqrt{2\delta_k - \delta_k^2}\right) - \ln(1-\delta_k) \\
\implies &\frac{1}{k}\cosh^{-1}(1/\epsilon) = \sqrt{2\delta_k} + \mathcal{O}(\delta_k) \\
\implies &k = \frac{\cosh^{-1}(1/\epsilon)}{\sqrt{2\delta_k}} + \mathcal{O}(1).
\end{aligned} \tag{37}$$

□

Proposition 7. Let $a \in]0,1[$, $b \in [a,1[$ and consider the sequence of polynomials $(\upsilon_{a,k})_{k\in\mathbb{N}}$. Define the sequence $(x_k(a,b))_{k\in\mathbb{N}} = \left(1 - \max \upsilon_{a,k}^{-1}(b)\right)_{k\in\mathbb{N}}$ and $\delta_k(a) = (T_{1/k}(1/a) - 1)/T_{1/k}(1/a)$. There exists a constant $c \in]0,1[$, independent of a and b, such that:

$$\frac{x_k(a,b)}{\delta_k(a)} \geq c\left(\frac{\log(1/b)}{\log(1/a)}\right)^2. \tag{38}$$

Proof. Let $m(k)$ be the smallest integer $m \in \mathbb{N}$ such that $\delta_m(a) \leq x_k(a,b)$. Note that $\upsilon_{a,k} \in \mathcal{C}(b, m(k)-1)$. Theorem 2 implies that $k \geq m(k)-1$. Also, Proposition 1 implies the existence of a constant $c_1 > 0$ such that:

$$m(k) - 1 \geq \frac{c_1 \log(1/b)}{\sqrt{\delta_{m(k)}(a)}} \geq \frac{c_1 \log(1/b)}{\sqrt{x_k(a,b)}}. \tag{39}$$

Proposition 1 also implies the existence of $c_2 > c_1$ such that $k \leq c_2 \log(1/a)/\sqrt{\delta_k}$. As a consequence,

$$\frac{c_1 \log(1/b)}{\sqrt{x_k(a,b)}} \leq \frac{c_2 \log(1/a)}{\sqrt{\delta_k(a)}} \implies \frac{x_k(a,b)}{\delta_k(a)} \geq c\left(\frac{\log(1/b)}{\log(1/a)}\right)^2, \tag{40}$$

where we defined $c = (c_1/c_2)^2$. □

Proposition 8. Let $a \in]0,1[$, $b \in [a,1[$, $\delta, \epsilon \in]0,1[$. There exists a polynomial $r_{a,b,\delta,\epsilon}$ of degree $\mathcal{O}\left(\frac{\log(1/\epsilon)\log(1/a)}{(b-a)\sqrt{\delta}}\right)$ such that:

- $r_{a,b,\delta,\epsilon}(x) \in [-1,1]$ for all $x \in [-1,1]$,
- $|r_{a,b,\delta,\epsilon}(x)| \leq \epsilon$ for all $x \in [-1, 1-\delta]$,
- $r_{a,b,\delta,\epsilon}(x) \geq 1-\epsilon$ for all $x \in [-1, 1 - c\delta\log(1/b)^2/\log(1/a)^2]$,

where c is the constant introduced in Proposition 7.

Proof. Let $q_{\frac{a+b}{2},b-a,\epsilon}$ be the polynomial of degree $\mathcal{O}\left(\frac{\log(1/\epsilon)}{b-a}\right)$ defined in Proposition 6 with center $(a+b)/2$, precision $(b-a)$ and error ϵ. Define the polynomial $r_{a,b,\delta,\epsilon} = q_{\frac{a+b}{2},b-a,\epsilon} \circ \upsilon_{a,k}$, with k, odd and minimal such that $\delta_k \leq \delta$, as in Theorem 2 and of degree $\mathcal{O}\left(\frac{\log(1/\epsilon)\log(1/a)}{(b-a)\sqrt{\delta}}\right)$. As the composition of two polynomials sending $[-1,1]$ to a subset of $[-1,1]$, the polynomial sends $[-1,1]$ to a subset of $[-1,1]$. If $x \in [-1, 1-\delta]$, then $\upsilon_{a,k}(x) \leq a$ so that $\left|q_{\frac{a+b}{2},b-a,\epsilon}(\upsilon_{a,k}(x))\right| \leq \epsilon$. If $x \in [-1, 1-c\delta\log(1/b)^2/\log(1/a)^2]$, then $\upsilon_{a,k}(x) \geq b$ and $q_{\frac{a+b}{2},b-a,\epsilon}(\upsilon_{a,k}(x)) \geq 1-\epsilon$. Summarizing, $q_{\frac{a+b}{2},b-a,\epsilon}$ satisfies the desired properties. □

The complementary polynomial of $A\upsilon_n$ may be computed with techniques described in [5] and may have a scaling factor β different from 1. Still, β is logarithmic in the degree of υ_n. Note also that once we know a lower bound for the degree of a polynomial with the properties given in Corollary 8, we can also compute it with a multi-interval Remez algorithm [7].

IV. PROPERTIES OF THE FLAT DISCRIMINANT

This section introduces the flat discriminant of a Markov chain. This operator comes to replace the curved discriminant introduced in Section II when the adjoint operator is not known. We study sequences of discriminants associated to the powers of a given ergodic Markov kernel. More precisely, we consider the rate at which these sequences $(D_j)_{j\geq 1}$ become similar to $(P^j)_{j\geq 1}$ or $\left((P^j + (P^\star)^j)/2\right)_{j\geq 1}$ (in the sense that they are equal up to a change of basis). In particular, we describe a phenomenon in which this similarity occurs long before the mixing time. We then give examples and intuitive conditions under which this phenomenon occurs. The results of Section III, applied to a flat discriminant satisfying the given properties, allow to sample from an approximation of the stationary distribution faster than the mixing time of the chain. Therefore, it significantly broadens the set of Markov chains for which a quantum algorithm offers a speedup over classical methods.

A. Generic properties

The present section goes over simple properties of the flat discriminant, starting by its definition.

Definition 15. Let P be a Markov transition matrix over state space $\mathbb{S}$ of size $|\mathbb{S}| = n$. Define its flat discriminant by its matrix elements:

$$\forall x, y \in \mathbb{S} : D(x, y) = \sqrt{P(x, y)P(y, x)}. \tag{41}$$

More generally, define the j-th order flat discriminant D_j as the flat discriminant of P^j, for any integer $j \in \mathbb{N}$. The leading eigenvalue of D_j is denoted by $\mu(j)$ and, if unique, the corresponding eigenvector $|\mu(j)\rangle$. $|\mu\rangle = |\mu(1)\rangle$ will be referred to as the most reversible distribution of the kernel P.

Since it arises from a projected unitary encoding, it is clear that the spectral norm of a flat discriminant is less than 1. The following proposition gives an independent proof.

Proposition 9. The eigenvalues of D are contained in $[-1, 1]$.

Proof. D being symmetric, its eigenvalues are on the real line. Also, its spectral norm is equal to its spectral radius. Moreover, D is similar to

$$E : \mathbb{S}^2 \to \mathbb{R}_+, (x, y) \mapsto \sqrt{P(x, y)P^\star(x, y)}. \tag{42}$$

The Cauchy-Schwarz inequality implies that:

$$\forall x \in \mathbb{S} : \sum_{y\in\mathbb{S}} |E(x, y)| \leq \sqrt{\sum_{y\in\mathbb{S}} P(x, y) \sum_{y\in\mathbb{S}} P^\star(x, y)} = 1. \tag{43}$$

As a consequence, E contracts the $\|\cdot\|_\infty$ norm. In particular, its spectral radius and that of D is less than 1. □

Corollary 3. If P is lazy, the eigenvalues of D are in $[0, 1]$.

Proof. If P is lazy, it can be written as $P = \frac{I+Q}{2}$ for some transition matrix Q. Let D be the flat discriminant of P and E be the flat discriminant of Q. Then, for each $x \in \mathbb{S}$, $D(x, x) = \frac{1+Q(x,x)}{2} = \frac{1+E(x,x)}{2} = \frac{I+E}{2}(x, x)$. If $x \neq y \in \mathbb{S}$:

$$D(x, y) = \sqrt{\frac{Q(x, y)}{2}\frac{Q(y, x)}{2}} = \frac{E(x, y)}{2} = \frac{I + E}{2}(x, y). \tag{44}$$

Therefore, $D = \frac{I+E}{2}$ and the property of the eigenvalues follow. □

The following Proposition 10 and Proposition 11 explain that $|\pi\rangle$ can only be an eigenvector of D if the lines of P and those of $P^\star$ have a constant overlap. Interestingly, the simple fact that 1 belongs to the spectrum of D implies that all the eigenvalues of the kernel and its discriminant are equal and that $|\pi\rangle$ is the most reversible distribution.

Proposition 10. Let P be an ergodic Markov kernel and D its flat discriminant. Then, $1 \in \sigma(D)$ if and only if P is reversible. In particular, if $1 \in \sigma(D)$, then $D = \mathcal{D}$.

Proof. Let $1 \in \sigma(D)$ with corresponding eigenvector $|\mu\rangle = \sum_{x\in\mathbb{S}} \sqrt{\mu(x)}\,|x\rangle$ for some probability distribution μ on $\mathbb{S}$. The eigenvector can always be chosen to be of this form because D has positive matrix elements. $|\mu\rangle$ satisfies:

$$\langle\mu|D|\mu\rangle = \sum_{x,y\in\mathbb{S}} \sqrt{\mu(x)P(x,y)P(y,x)\mu(y)} = 1. \tag{45}$$

However, $(x,y) \mapsto \mu(x)P(x,y)$ and $(x,y) \mapsto \mu(y)P(y,x)$ form valid probability distributions on $\mathbb{S}^2$. If they have overlap 1, they must be equal. Therefore, P is μ-reversible. In particular, $\mu P = \mu$. Since we assume uniqueness of the left eigenvector of P with eigenvalue 1, $\mu = \pi$. Conversely, if P is reversible, then $D = \mathcal{D}$ and $\sigma(D) = \sigma(\mathcal{D}) = \sigma(P) \ni 1$.

Proposition 11 below is also easy to see and makes use of the definition of an irreducible matrix.

Definition 16. A matrix A is irreducible if and only if its associated graph is strongly connected.

Proposition 11. $|\pi\rangle$ is an eigenvector of D with eigenvalue $0 \le \lambda \le 1$ if and only if $\langle P(x,\cdot)|P^\star(x,\cdot)\rangle = \lambda$, for each $x \in \mathbb{S}$. If in addition D is irreducible, then $\sigma(D) \subset [-\lambda, \lambda]$ and the λ-eigenspace is one-dimensional and generated by $|\pi\rangle$.

Proof. The first claim is trivial. The second is an immediate corollary of the Perron-Frobenius Theorem 6. □

The following proposition is trivial, but useful when P and $P^\star$ do not mix at the same rate. See subsection V A for an example.

Proposition 12. The flat discriminant of P and the flat discriminant of $P^\star$ are equal.

Proof. Let $x, y \in \mathbb{S}$, D the flat discriminant of P and $D^\star$ that of $P^\star$. Then,

$$D(x,y) = \sqrt{P(x,y)P(y,x)} = \sqrt{\frac{\pi(y)P^\star(y,x)}{\pi(x)}\frac{\pi(x)P^\star(x,y)}{\pi(y)}} = \sqrt{P^\star(x,y)P^\star(y,x)} = D^\star(x,y). \tag{46}$$

□

Let us relate the overlap between the stationary distribution $|\pi\rangle$ and the most reversible distribution $|\mu\rangle$ to the spectrum of D. Proposition 13 is trivial but the proof is given for completeness.

Proposition 13. Let $\mu, \lambda_1, ..., \lambda_{n-1}$ be the eigenvalues of D, in decreasing order. Denote by $|\mu\rangle, |\lambda_1\rangle, ..., |\lambda_{n-1}\rangle$ the corresponding eigenvectors. Then,

$$|\langle\pi|\mu\rangle|^2 \ge \frac{\langle\pi|D|\pi\rangle - \lambda_1}{\mu - \lambda_1}. \tag{47}$$

Proof. Decompose $|\pi\rangle$ in the orthonormal basis of eigenvectors of D as $|\pi\rangle = |\mu\rangle\langle\mu|\pi\rangle + \sum_{i=1}^{n-1} c_i |\lambda_i\rangle$. Then, compute:

$$\begin{aligned}\langle\pi|D|\pi\rangle &= \mu|\langle\pi|\mu\rangle|^2 + \sum_{i=1}^{n-1}\lambda_i|c_i|^2\\ &\le \mu|\langle\pi|\mu\rangle|^2 + \lambda_1\sum_{i=1}^{n-1}|c_i|^2\\ &= (\mu-\lambda_1)|\langle\pi|\mu\rangle|^2 + \lambda_1.\end{aligned} \tag{48}$$

□

Using the same arguments, one can show the more intuitive Proposition 14.

Proposition 14. Let $|\mu\rangle$ be the leading eigenvector of D. Then,

$$\langle\pi|\mu\rangle^2 \ge 1 - \frac{1 - \langle\pi|D|\pi\rangle}{\gamma(P)}, \tag{49}$$

where $\gamma(P)$ is the Poincaré constant of P.

Proof. Let $\mathcal{D}_A$ be the curved discriminant of $(P+P^\star)/2$. Repeating the arguments in the proof of Proposition 13 with the orthonormal eigenbasis of $\mathcal{D}_A$ and $|\mu\rangle$ instead of $|\pi\rangle$ yields:

$$\langle\mu|\mathcal{D}_A|\mu\rangle \leq \gamma(P)|\langle\pi|\mu\rangle|^2 + 1 - \gamma(P). \tag{50}$$

Applying the inequality $\sqrt{ab} \leq (a+b)/2$ (valid for all positive numbers a and b) to the matrix elements of D shows that $D \leq \mathcal{D}_A$ element-wise. Thus, $\langle\mu|\mathcal{D}_A|\mu\rangle \geq \langle\mu|D|\mu\rangle \geq \langle\pi|D|\pi\rangle$ and the claim is proven. □

To quantify how close P is to its equilibrium, the quantity $\max_{x\in\mathbb{S}} d_{TV}(P(x,\cdot),\pi)$ is often used. Indeed, it presents relevant properties such as being decreasing and submultiplicative. In the present study, the overlap between probability distributions appears more often than the total variation distance separating them. Proposition 15 shows that the law of the chain starting from $x \in \mathbb{S}$ has increasing overlap with $|\pi\rangle$. This implies, as stated in Corollary 4, that the maximum Hellinger distance is decreasing.

Proposition 15. For each $x \in \mathbb{S}$, $\langle P^j(x,\cdot)|\pi\rangle$ is increasing with $j \in \mathbb{N}$.

Proof. Let $x \in \mathbb{S}$ and $j \geq 0$. According to the Cauchy-Schwarz inequality:

$$\begin{aligned}\langle P^{j+1}(x,\cdot)|\pi\rangle &= \sum_{y\in\mathbb{S}} \sqrt{P^{j+1}(x,y)\pi(y)} \\ &\geq \sum_{y,z\in\mathbb{S}} \sqrt{P^j(x,z)P(z,y)\pi(z)P(z,y)} \\ &= \sum_{y,z\in\mathbb{S}} P(z,y)\sqrt{P^j(x,z)\pi(z)} \\ &= \sum_{z\in\mathbb{S}} \sqrt{P^j(x,z)\pi(z)} \\ &= \langle P^j(x,\cdot)|\pi\rangle\,.\end{aligned} \tag{51}$$

□

Corollary 4. The maximum Hellinger distance $\max_{x\in\mathbb{S}} \sqrt{1-\langle P^j(x,\cdot)|\pi\rangle}$ is decreasing with $j \in \mathbb{N}$.

Interestingly, the π-average of the total variation distance to π (over the stating states sampled from π) converges significantly faster to 0 than the mixing time in the worst case. The precise result is stated in Proposition 16, which follows from Lemma 2.

Lemma 2. Let P be an ergodic Markov kernel and $t \in \mathbb{N}$. Then,

$$\forall x \in \mathbb{S} : d_{TV}(P^t(x,\cdot),\pi) \leq \frac{1}{2}\left(\frac{1}{\pi(x)} - 1\right)^{1/2} e^{-\gamma(P^\star P)t/2}. \tag{52}$$

If in addition P is lazy, then

$$\forall x \in \mathbb{S} : d_{TV}(P^t(x,\cdot),\pi) \leq \frac{1}{2}\left(\frac{1}{\pi(x)} - 1\right)^{1/2} e^{-\gamma(P)t/2}. \tag{53}$$

Lemma 2 is well-known but the proof is given for completeness.

Proof. Using the Cauchy-Schwarz inequality:

$$\forall x \in \mathbb{S} : d_{TV}(P^t(x,\cdot),\pi) \leq \frac{1}{2}\left(\sum_{y\in\mathbb{S}} \pi(y)\left|\frac{P^t(x,y)}{\pi(y)} - 1\right|^2\right)^{1/2} \leq \frac{1}{2}\mathrm{Var}((P^\star)^t f_x)^{1/2}, \tag{54}$$

where $f_x : \mathbb{S} \to \mathbb{R}$, $y \mapsto \delta_x(y)/\pi(y)$. Using the definition of the Poincaré constant and the fact that $\mathrm{Var}(f_x) = \frac{1}{\pi(x)} - 1$:

$$\mathrm{Var}((P^\star)^t f_x) \leq \mathrm{Var}(f_x)(1-\gamma(P^\star P))^t \leq \left(\frac{1}{\pi(x)} - 1\right) e^{-\gamma(P^\star P)t}. \tag{55}$$

If P is lazy, the above inequality holds with $\gamma(P)$ instead of $\gamma(P^\star P)$. □

Proposition 16. Let $\epsilon > 0$ and $|\mathbf{1}\rangle = \frac{1}{n}\sum_{x\in\mathbb{S}}|x\rangle$. If $j \geq \frac{2\ln(\sqrt{n}\langle\pi|\mathbf{1}\rangle/\epsilon)}{\gamma(P^\star P)}$, then:

$$\mathbb{E}_\pi\left[d_{TV}(P^j(x,\cdot),(P^\star)^j(x,\cdot))\right] \leq \epsilon. \tag{56}$$

If P is lazy and $j \geq \frac{2\ln(\sqrt{n}\langle\pi|\mathbf{1}\rangle/\epsilon)}{\gamma(P)}$, then

$$\mathbb{E}_\pi[d_{TV}(P^j(x,\cdot),(P^\star)^j(x,\cdot))] \leq \epsilon. \tag{57}$$

Proof. Using the triangle inequality and Lemma 2:

$$\begin{aligned}
\mathbb{E}_\pi\left[d_{TV}(P^j(x,\cdot),(P^\star)^j(x,\cdot))\right] &= \frac{1}{2}\sum_{x,y\in\mathbb{S}}\pi(x)|P^j(x,y)-(P^\star)^j(x,y)| \\
&= \frac{1}{2}\sum_{x,y\in\mathbb{S}}|\pi(x)P^j(x,y)-\pi(y)P^j(y,x)| \\
&\leq \frac{1}{2}\sum_{x,y\in\mathbb{S}}\pi(x)|P^j(x,y)-\pi(y)| + \frac{1}{2}\sum_{x,y\in\mathbb{S}}\pi(y)|\pi(x)-P^j(y,x)| \\
&= 2\mathbb{E}_\pi\left[d_{TV}(P^j(x,\cdot),\pi)\right] \\
&\leq \mathbb{E}_\pi\left[e^{-\gamma(P^\star P)j/2}\sqrt{\frac{1}{\pi(x)}-1}\right] \\
&\leq \sum_{x\in\mathbb{S}}\sqrt{\pi(x)}e^{-\gamma(P^\star P)j/2} \\
&= \sqrt{n}\,\langle\pi|\mathbf{1}\rangle\,e^{-\gamma(P^\star P)j/2}.
\end{aligned} \tag{58}$$

Solving for the right-hand side to be less than ϵ proves the claim without the lazyness hypothesis. If P is lazy, the same reasoning proves the second claim. ☐

B. ϵ-mixed kernels

Let us now state properties of the flat discriminant of a kernel that is almost mixed. More precisely, for $0 < \epsilon \leq 1/n$, the flat discriminant of $P^{\tau(\epsilon)}$ has large leading eigenvalue, large gap and large overlap between its leading eigenvector and $|\pi\rangle$. If $1/n < \epsilon < 1/4$, replacing $P^{\tau(\epsilon)}$ by $P^{m\tau(\epsilon)}$ with $m \in \mathcal{O}(\log(n))$ yields a discriminant with the same properties. Let us start with a lower bound on $\langle\pi|D|\pi\rangle$ that depends only on the maximum total variation distance to stationary among all localized starting states.

Lemma 3. Let $d = \max_{x\in\mathbb{S}} d_{TV}(P(x,\cdot),\pi)$. Then,

$$\langle\pi|D|\pi\rangle \geq 1-2d. \tag{59}$$

Proof. Define a probability measure ν on $\mathbb{S}^2$ by $\nu(x,y) = \pi(x)P(x,y)$ for each $x,y\in\mathbb{S}$. Using Proposition 35 for $p=\nu$ and $q=\nu^T$ and the triangle inequality:

$$\begin{aligned}
\langle\pi|D|\pi\rangle &\geq 1-\frac{1}{2}\sum_{x,y\in\mathbb{S}}|\pi(x)P(x,y)-\pi(y)P(y,x)| \\
&\geq 1-\sum_{x,y\in\mathbb{S}}\pi(x)|P(x,y)-\pi(y)| \\
&\geq 1-2d.
\end{aligned} \tag{60}$$

☐

The above lemma ensures that the leading eigenvalue is close to 1. The following proposition will use the fact that the spectral norm between the discriminant and the projector $|\pi\rangle\langle\pi|$ is small to show that all the other eigenvalues of D are small. $\langle\pi|D|\pi\rangle$ being large, the overlap of the stationary and the most reversible distribution will necessarily be large also.

Proposition 17. Assume that $\max_{x\in\mathbb{S}} d_{TV}(P(x,\cdot),\pi) \leq \epsilon$ for some $0 \leq \epsilon < 1/n$. Then, $\langle\pi|D|\pi\rangle \geq 1-2\epsilon$, $|\langle\mu|\pi\rangle|^2 \geq 1-2\epsilon+\mathcal{O}(\epsilon^{3/2})$ and all eigenvalues of D except the largest are smaller than $2\sqrt{2}\sqrt{\epsilon}$.

Proof. By Lemma 3, $\langle\pi|D|\pi\rangle \geq 1-2\epsilon$. Let us upper bound the Frobenius norm-induced distance between D and $|\pi\rangle\langle\pi|$.

$$\begin{aligned}
\|D-|\pi\rangle\langle\pi|\|_F^2 &= \sum_{x,y\in\mathbb{S}} \left|\sqrt{P(x,y)P(y,x)}-\sqrt{\pi(x)\pi(y)}\right|^2 \\
&= \mathrm{Tr}\left(P^2\right)+1-2\langle\pi|D|\pi\rangle \\
&= \sum_{x,y\in\mathbb{S}} (P(x,y)-\pi(y))(P(y,x)-\pi(x))+2\pi(y)(P(y,x)-\pi(x))+\pi(y)\pi(x)+1-2\langle\pi|D|\pi\rangle \\
&= \sum_{x,y\in\mathbb{S}} (P(x,y)-\pi(y))(P(y,x)-\pi(x))+2-2\langle\pi|D|\pi\rangle \qquad (61)\\
&\leq 2\epsilon\sum_{x,y\in\mathbb{S}} |P(x,y)-\pi(y)|+2-2\langle\pi|D|\pi\rangle \\
&\leq 4n\epsilon^2+2-2\langle\pi|D|\pi\rangle \\
&\leq 4n\epsilon^2+4\epsilon \\
&\leq 8\epsilon.
\end{aligned}$$

This implies in particular that $\|D-|\pi\rangle\langle\pi|\| \leq 2\sqrt{2}\sqrt{\epsilon}$. As a consequence of Theorem 7, the eigenvalues of D that are not $2\sqrt{2}\sqrt{\epsilon}$ close to 1 must be less than $2\sqrt{2}\sqrt{\epsilon}$ in magnitude. Proposition 13 gives a lower bounds on the overlap between $|\mu\rangle$ and $|\pi\rangle$:

$$|\langle\mu|\pi\rangle|^2 \geq \frac{1-2\epsilon-2\sqrt{2}\sqrt{\epsilon}}{1-2\sqrt{2}\sqrt{\epsilon}} = 1-2\epsilon+\mathcal{O}(\epsilon^{3/2}). \qquad (62)$$

□

Corollary 5. Let $0<\epsilon<1/4$ and $t=\min(\tau(\epsilon),\tau^\star(\epsilon))$ where $\tau(\epsilon)$ is the mixing time of P and $\tau^\star(\epsilon)$ is the mixing time of $P^\star$. It is possible to implement the reflection $2|\mu(\tilde{t})\rangle\langle\mu(\tilde{t})|-1$ up to spectral norm error $\eta>0$ with $\mathcal{O}(\log(1/\eta))$ uses of R the Szegedy quantum walk operator associated with $P^{\tilde{t}}$, where $\tilde{t}=t\lceil\log(n)\rceil$. Moreover, $\langle\mu(\tilde{t})|\pi\rangle \geq 1-(2\epsilon)^{\lceil\log(n)\rceil}+\mathcal{O}\left((2\epsilon)^{3\lceil\log(n)\rceil/2}\right)$.

Proof. For each integer $m\in\mathbb{N}$, define $d(m)=\max_{x\in\mathbb{S}} d_{TV}(P^m(x,\cdot),\pi)$. This quantity satisfies $d(l+m)\leq 2d(l)d(m)$ for each $l,m\in\mathbb{N}$ (see for example [8]). As a consequence, $d(\tilde{t}) \leq (2\epsilon)^{\lceil\log(n)\rceil}/2 \leq 2^{-1-\lceil\log(n)\rceil} < 1/n$. Lemma 3 implies that $\langle\mu(\tilde{t})|\pi\rangle \geq 1-(2\epsilon)^{\lceil\log(n)\rceil}+\mathcal{O}\left((2\epsilon)^{3\lceil\log(n)\rceil/2}\right)$. □

C. Relation between the spectra of the kernel and its discriminant

This section aims at upper bounding the spectral norm of $D-\mathcal{D}$. The Bauer-Fike Theorem 7 translates into an upper bound on the second largest eigenvalue of D in terms of the absolute spectral gap of P and $\langle\pi|D|\pi\rangle$. Compared to Proposition 3 of the previous section, the following proposition is interesting long before the mixing time.

Proposition 18. Define $c=\max_{x,y\in\mathbb{S}}\sqrt{\frac{P(x,y)}{\pi(y)}}$. Then,

$$\|\mathcal{D}-D\| \leq c\sqrt{2(1-\langle\pi|D|\pi\rangle)}. \qquad (63)$$

Proof. Let $x\in\mathbb{S}$ and $f:\mathbb{S}\to\mathbb{C}$. Then,

$$\begin{aligned}
|(\mathcal{D}-D)f(x)| &\leq \sum_{y\in\mathbb{S}}\sqrt{\frac{\pi(x)}{\pi(y)}}\left|P(x,y)-\sqrt{P(x,y)P^\star(x,y)}\right||f(y)| \\
&\leq c\sum_{y\in\mathbb{S}}\sqrt{\pi(x)}\left|\sqrt{P(x,y)}-\sqrt{P^\star(x,y)}\right||f(y)|
\end{aligned} \qquad (64)$$

Defining $H : \mathbb{S}^2 \to \mathbb{R}$ by $H(x,y) = \sqrt{\pi(x)} \left| \sqrt{P(x,y)} - \sqrt{P^\star(x,y)} \right|$, the above equation can be written $|(\mathcal{D}-D)f(x)| \leq cH|f|(x)$ for all $x \in \mathbb{S}$. As a consequence, $\|(\mathcal{D}-D)f\|_2 \leq c\|H\|\|f\|_2$. Recall that the spectral norm is smaller than the Frobenius norm and that $\|H\|_F = \sqrt{2(1-\langle\pi|D|\pi\rangle)}$:

$$\|(\mathcal{D}-D)f\|_2 \leq c\|H\|\|f\|_2 \leq c\sqrt{2(1-\langle\pi|D|\pi\rangle)}\|f\|_2. \tag{65}$$

As a consequence, $\|\mathcal{D}-D\| \leq c\sqrt{2(1-\langle\pi|D|\pi\rangle)}$. □

Using the bound from Proposition 18 and perturbation theory leads to the following corollary.

Corollary 6. Let δ be the absolute spectral gap of P. If $c\sqrt{2(1-\langle\pi|D|\pi\rangle)} < \delta/2$, then the second largest eigenvalue λ of D satisfies:

$$|\lambda| \leq 1 - \delta + c\sqrt{2(1-\langle\pi|D|\pi\rangle)}, \tag{66}$$

where δ is the absolute spectral gap of P. Moreover, the overlap between between the most reversible distribution μ of D and the stationary distribution π satisfies:

$$\langle\mu|\pi\rangle^2 \geq 1 - \frac{1-\langle\pi|D|\pi\rangle}{\delta - c\sqrt{2(1-\langle\pi|D|\pi\rangle)}}. \tag{67}$$

Also, note that if we write $c(t) = \max_{x,y\in\mathbb{S}} \sqrt{P^t(x,y)/\pi(y)}$ for each $t \in \mathbb{N}$ and $x, z \in \mathbb{S}$ such that $c(t+1)^2 = \frac{P^{t+1}(x,z)}{\pi(z)}$:

$$c(t+1)^2 = \frac{P^{t+1}(x,z)}{\pi(z)} = \sum_{y\in\mathbb{S}} \frac{P(x,y)P^t(y,z)}{\pi(z)} \leq c(t)^2 \sum_{y\in\mathbb{S}} P(x,y) = c(t)^2. \tag{68}$$

Also, $\lim_{t\to\infty} c(t) = 1$. In words, $c(t)$ decreases to 1. It takes the maximal possible value of $1/\sqrt{\pi_\star}$ only if, for some state $x \in \mathbb{S}$, the chain goes to $y = \text{argmin}_{z\in\mathbb{S}}\pi(z)$ with probability 1. Also, the inequality used in Equation 68 is only sharp if $P^t(y,z)/\pi(z)$ is close from $c(t)^2$ for each $y \in \mathbb{S}$ such that $P(x,y) > 0$. If $c(t)$ is much larger than 1, this constraint cannot be satisfied because $\sum_z P^t(y,z) = 1$ for each $y \in \mathbb{S}$.

D. Relation between the spectra of the additive reversibilization of the kernel and its discriminant

This section compares the spectra of D and the additive reversibilization of the kernel $P_A = (P+P^\star)/2$. Proposition starts by upper bounding the norm of $D - \mathcal{D}_A$ using a quantity $\omega \in [0,1]$. Consider a kinetic process in a potential. P and $P^\star$ tend to agree on the direction to take where the potential is very steep, and to disagree on saddle points of the potential. Starting from any state $x \in \mathbb{S}$, $P_A(x,\cdot)$ will typically be an equal weight superposition of two distributions, each centered on a local minima of the potential. Starting from the minima of probability, both P and $P^\star$ tend to stay in a neighborhood of the minima and to have rapidly increasing overlap. As a consequence, ω tends to be close to 1.

Proposition 19. Let $P_A = (P+P^\star)/2$ be the additive reversibilization of P and $\mathcal{D}_A$ its discriminant. Let $w = \min_{x\in\mathbb{S}} \sum_{y\in\mathbb{S}} P_A(x,y) \langle P(y,\cdot)|P^\star(y,\cdot)\rangle$. Then,

$$\|\mathcal{D}_A - D\| \leq \sqrt{1-w}. \tag{69}$$

Proof. Because $\mathcal{D}_A - D$ is symmetric: $\|\mathcal{D}_A - D\| = \sqrt{\|(\mathcal{D}_A - D)^2\|}$. The spectral norm of $(\mathcal{D}_A - D)^2$ is equal to its spectral radius. Moreover, $(\mathcal{D}_A - D)^2$ is similar to

$$H : \mathbb{S}^2 \to \mathbb{R}_+, (x,z) \mapsto \sum_{y\in\mathbb{S}} \left(P_A(x,y) - \sqrt{P(x,y)P^\star(x,y)}\right)\left(P_A(y,z) - \sqrt{P(y,z)P^\star(y,z)}\right). \tag{70}$$

In particular, the spectral radius of $(\mathcal{D}_A - D)^2$ is equal to the spectral radius of H. H having only positive coefficients, its spectral radius is upper bounded by $\max_{x\in\mathbb{S}} \sum_{y\in\mathbb{S}} H(x,y)$. However,

$$\begin{aligned}
\forall x \in \mathbb{S} : \sum_{z\in\mathbb{S}} H(x,z) &= \sum_{y,z\in\mathbb{S}} \left(P_A(x,y) - \sqrt{P(x,y)P^\star(x,y)}\right)\left(P_A(y,z) - \sqrt{P(y,z)P^\star(y,z)}\right) \\
&\leq \sum_{y,z\in\mathbb{S}} P_A(x,y)\left(P_A(y,z) - \sqrt{P(y,z)P^\star(y,z)}\right) \\
&= 1 - \sum_{y\in\mathbb{S}} P_A(x,y)\langle P(y,\cdot)|P^\star(y,\cdot)\rangle \\
&\leq 1 - w.
\end{aligned} \tag{71}$$

Therefore, $\|\mathcal{D}_A - D\| \leq \sqrt{1-w}$. □

Note that π is a linear combination of the lines of P_A. As such, $w \leq \langle\pi|D|\pi\rangle$. When ω is close enough from 1, the Bauer-Fike Theorem 7 implies the following proposition.

Proposition 20. Let γ_A be the spectral gap of P. If $\sqrt{1-\omega} < \gamma_A/2$, then the largest eigenvalue of D is larger than ω, the second largest is smaller than $1-\gamma_A+\sqrt{1-\omega}$ and

$$\langle\pi|\mu\rangle^2 \geq 1 - \frac{1-\omega}{\gamma_A - \sqrt{1-\omega}}. \tag{72}$$

E. Random walks on finite groups

When the Markov chain presents additional symmetries the distance between the eigenvalues of the flat discriminant and those of the additive reversibilisation of the chain $(P+P^\star)/2$ are $1-\langle\pi|D|\pi\rangle$ close. This improves on more general upper bounds in $\mathcal{O}\left(\sqrt{1-\langle\pi|D|\pi\rangle}\right)$. Let us start by defining random walks on finite groups.

Definition 17. Consider a finite group $(\mathbb{S},\circ)$ and a probability distribution ν on $\mathbb{S}$. Let $(Z_t)_{t\geq 1}$ be independent and identically distributed random variables with law ν, and consider the process $X=(X_t)_{t\geq 1}$ with initial condition $X_0 \in \mathbb{S}$ defined by:

$$X_t = Z_t \circ Z_{t-1} \circ \ldots \circ Z_1 \circ X_0. \tag{73}$$

Then X is a Markov chain on $\mathbb{S}$ called the random walk on $(\mathbb{S},\circ)$ with increment law ν. Its transition kernel is given for all $x,y\in\mathbb{S}$ by $P(x,y)=\nu\left(y\circ x^{-1}\right)$.

The following proposition is trivial but useful to consider powers of a random walk on groups instead of the walk itself.

Proposition 21. Assume that P is a random walk on the finite group $(\mathbb{S},\circ)$. Then, for each $j\geq 2$, P^j is a random walk on the finite group $(\mathbb{S},\circ)$.

Proof. Assume that for some $j\geq 1$, P^j is a random walk on $(\mathbb{S},\circ)$. By definition, there exists a measure ν_j on $\mathbb{S}$ such that $P^j(x,y)=\nu_j\left(y\circ x^{-1}\right)$ for all $x,y\in\mathbb{S}$. Then,

$$\begin{aligned}\forall x,z\in\mathbb{S}: P^{j+1}(x,z) &= \sum_{y\in\mathbb{S}} \nu_j\left(y\circ x^{-1}\right)\nu\left(z\circ y^{-1}\right)\\ &= \sum_{w\in\mathbb{S}} \nu_j(w)\nu\left(z\circ (w\circ x)^{-1}\right)\\ &= \sum_{w\in\mathbb{S}} \nu_j(w)\nu\left(z\circ x^{-1}\circ w^{-1}\right).\end{aligned} \tag{74}$$

Define for each $x\in\mathbb{S}$ the measure ν_{j+1} by:

$$\nu_{j+1}(x) = \sum_{w\in\mathbb{S}} \nu_j(w)\nu\left(x\circ w^{-1}\right). \tag{75}$$

Then $P^{j+1}(x,z)=\nu_{j+1}\left(z\circ x^{-1}\right)$ for each $x,z\in\mathbb{S}$ and P^{j+1} is a random walk on $(\mathbb{S},\circ)$. By induction, all powers of P are random walks on the finite group $(\mathbb{S},\circ)$. □

In order to use perturbation theory arguments, let us compute the spectral norm of $D-\mathcal{D}_A$.

Proposition 22. Assume that P is a random walk on a group $(\mathbb{S},\circ)$. Then, $\langle\pi|\mu\rangle = 1$ and $\|\mathcal{D}_A - D\| = 1-\langle\pi|D|\pi\rangle$.

Proof. Let $x\in\mathbb{S}$:

$$\begin{aligned}\sum_{y\in\mathbb{S}} D(x,y) &= \sum_{y\in\mathbb{S}} \sqrt{\nu\left(y\circ x^{-1}\right)\nu\left(x\circ y^{-1}\right)}\\ &= \sum_{z\in\mathbb{S}} \sqrt{\nu(z)\nu\left(z^{-1}\right)}.\end{aligned} \tag{76}$$

The sum of the lines of D being constant, its leading eigenvector is the uniform distribution. However, $|\pi\rangle$ is also the uniform distribution for random walk on groups. Let again $x \in \mathbb{S}$. Then,

$$\begin{aligned}
\sum_{y\in\mathbb{S}} |(\mathcal{D}_A - D)(x,y)| &= \frac{1}{2}\sum_{y\in\mathbb{S}} P(x,y) + P^\star(x,y) - 2\sqrt{P(x,y)P^\star(x,y)} \\
&= 1 - \frac{1}{n}\sum_{z,y\in\mathbb{S}} \sqrt{P(z,y)P(y,z)} \\
&= 1 - \langle\pi|D|\pi\rangle\,.
\end{aligned} \tag{77}$$

$\mathcal{D}_A - D$ being symmetric, its spectral norm is equal to its spectral radius and upper bounded by $1 - \langle\pi|D|\pi\rangle$. The uniform vector being a right eigenvector of $\mathcal{D}_A - D$ with eigenvalue $1 - \langle\pi|D|\pi\rangle$, $\|\mathcal{D}_A - D\| = 1 - \langle\pi|D|\pi\rangle$. □

This time, the Bauer-Fike Theorem 7 yields precise estimates on the location of the eigenvalues of D. Recall that the most reversible distribution is equal to the stationary distribution and that $\langle\pi|D|\pi\rangle = \langle P(0,\cdot)|P^T(0,\cdot)\rangle$ is easily computed. Further results will show how to use this information and the results from Section II to prepare the stationary distribution efficiently.

F. Flat discriminant and quasi-stationary distributions

If the state space can be decomposed as the union of regions within which fast local convergence occurs and which are long to escape, then $\langle\pi|D_j|\pi\rangle$ rapidly approaches 1 as $j \to \infty$. This idea is made rigorous using the concept of quasi-stationary distribution [9].

Definition 18. Let $(X_t)_{t\in\mathbb{N}}$ be a Markov chain with state space $E \cup \partial$ (with $\partial \cap E = \emptyset$) which is absorbed at ∂ (i.e. $\mathbb{P}_\partial(X_1 = \partial) = 1$). A quasi-stationary distribution is a probability measure ν on E such that:

$$\forall t \in \mathbb{N}, \forall A \subset E : \nu(X_t \in A | t < \tau_\partial) = \nu(A), \tag{78}$$

where $\tau_\partial = \inf\{t \geq 0, X_t \in \partial\}$ is the absorption time of X.

In practice, the state space may be written as $\mathbb{S} = \cup_{i=1}^m E_i \cup \partial$ for some $m \in \mathbb{N}$ and possess quasi-stationary distributions in each of the E_i (with complementary set $\partial_i = \cup_{j=1,j\neq i}^m E_j \cup \partial$, absorption time τ_{∂_i} and stationary distribution ν_i). $E = \cup_{i=1}^m E_i$ is a disjoint union.

Proposition 23. The following inequality holds for any integer $j \geq 1$:

$$\langle\pi|D_j|\pi\rangle \geq \pi(E) \min_{1\leq i\leq m} \min_{x\in E_i} \mathbb{P}_x(j < \tau_{\partial_i}) \min_{1\leq i\leq m} \left(1 - 2\mathbb{E}_{\pi_{|E_i}}\left[d_{TV}(\mathbb{P}_x(X_j = \cdot | j < \tau_{\partial_i}), \nu_i)\right] - d_{TV}\left(\pi_{|E_i} \otimes \nu_i, \nu_i \otimes \pi_{|E_i}\right)\right). \tag{79}$$

Proof. Expand:

$$\begin{aligned}
\langle\pi|D_j|\pi\rangle &\geq \sum_{i=1}^m \sum_{x,y\in E_i} \sqrt{\pi(x)\mathbb{P}_x(X_j = y|j < \tau_{\partial_i})\mathbb{P}_x(j < \tau_{\partial_i})\pi(y)\mathbb{P}_y(X_j = x|j < \tau_{\partial_i})\mathbb{P}_y(j < \tau_{\partial_i})} \\
&\geq \min_{1\leq i\leq m} \min_{x\in E_i} \mathbb{P}_x(j < \tau_{\partial_i}) \sum_{i=1}^m \sum_{x,y\in E_i} \sqrt{\pi(x)\mathbb{P}_x(X_j = y|j < \tau_{\partial_i})\pi(y)\mathbb{P}_y(X_j = x|j < \tau_{\partial_i})} \\
&= \min_{1\leq i\leq m} \min_{x\in E_i} \mathbb{P}_x(j < \tau_{\partial_i}) \sum_{i=1}^m \pi(E_i) \sum_{x,y\in E_i} \sqrt{\frac{\pi(x)\mathbb{P}_x(X_j = y|j < \tau_{\partial_i})\pi(y)\mathbb{P}_y(X_j = x|j < \tau_{\partial_i})}{\pi(E_i)^2}}.
\end{aligned} \tag{80}$$

The right-hand side is the overlap between two probability distributions on the edges of E_i: $F_i : (x,y) \mapsto \frac{\pi(x)}{\pi(E_i)}\mathbb{P}_x(X_j = y|j < \tau_{\partial_i})$ and its transpose F_i^T. As a consequence for each $1 \leq i \leq m$, Proposition 35 and the

triangle inequality imply:

$$\begin{aligned}
&\sum_{x,y\in E_i}\sqrt{\frac{\pi(x)\mathbb{P}_x(X_j=y|j<\tau_{\partial_i})\pi(y)\mathbb{P}_y(X_j=x|j<\tau_{\partial_i})}{\pi(E_i)^2}}\\
&\geq 1-\frac{1}{2}\sum_{x,y\in E_i}\left|\frac{\pi(x)\mathbb{P}_x(X_j=y|j<\tau_{\partial_i})}{\pi(E_i)}-\frac{\pi(y)\mathbb{P}_y(X_j=x|j<\tau_{\partial_i})}{\pi(E_i)}\right|\\
&=1-\frac{1}{2}\sum_{x,y\in E_i}\left|\frac{\pi(x)\mathbb{P}_x(X_j=y|j<\tau_{\partial_i})}{\pi(E_i)}-\frac{\pi(x)\nu_i(y)}{\pi(E_i)}\right.\\
&\left.+\frac{\pi(x)\nu_i(y)}{\pi(E_i)}-\frac{\pi(y)\nu_i(x)}{\pi(E_i)}+\frac{\pi(y)\nu_i(x)}{\pi(E_i)}-\frac{\pi(y)\mathbb{P}_y(X_j=x|j<\tau_{\partial_i})}{\pi(E_i)}\right|\\
&\geq 1-2\mathbb{E}_{\pi_{|E_i}}\left[d_{TV}(\mathbb{P}_x(X_j=\cdot|j<\tau_{\partial_i}),\nu_i)\right]-d_{TV}\left(\pi_{|E_i}\otimes\nu_i,\nu_i\otimes\pi_{|E_i}\right).
\end{aligned} \tag{81}$$

□

Proposition 23 is useful if it is reasonable to assume that, with j much smaller than the mixing time, each of the factors

- $\pi(E)$,
- $\min_{1\leq i\leq m}\min_{x\in E_i}\mathbb{P}_x(j<\tau_{\partial_i})$ and
- $\min_{1\leq i\leq m}\left(1-2\max_{x\in E_i}d_{TV}(\mathbb{P}_x(X_j=\cdot|j<\tau_{\partial_i}),\nu_i)-d_{TV}\left(\pi_{|E_i}\otimes\nu_i,\nu_i\otimes\pi_{|E_i}\right)\right)$

are large. If each of the factors is greater than $1-\epsilon$, then their product is greater than $1-3\epsilon+\mathcal{O}(\epsilon^2)$. Examples where these hypothesis are (numerically) verified are given in Section V. A similar statement involving the Hellinger distance is given by Proposition 24. The bound is often tighter since the involved distances appear through their square, but less intuitive from a probabilistic point of view.

Proposition 24. The following inequality holds for any integer $j\geq 1$:

$$\langle\pi|D_j|\pi\rangle\geq\min_{1\leq i\leq m}\min_{x\in E_i}\mathbb{P}_x(j<\tau_{\partial_i})\sum_{i=1}^m\pi(E_i)M_i, \tag{82}$$

where M_i is defined for each $1\leq i\leq m$ by:

$$1-M_i=8d_H\left(F_i,\pi_{|E_i}\otimes\nu_i\right)^2+2d_H\left(\pi_{|E_i}\otimes\nu_i,\nu_i\otimes\pi_{|E_i}\right)^2. \tag{83}$$

Proof. Starting as in the proof of Proposition 23,

$$\langle\pi|D_j|\pi\rangle\geq\min_{1\leq i\leq m}\min_{x\in E_i}\mathbb{P}_x(j<\tau_{\partial_i})\sum_{i=1}^m\pi(E_i)\sum_{x,y\in E_i}\sqrt{\frac{\pi(x)\mathbb{P}_x(X_j=y|j<\tau_{\partial_i})\pi(y)\mathbb{P}_y(X_j=x|j<\tau_{\partial_i})}{\pi(E_i)^2}}. \tag{84}$$

For each $1\leq i\leq m$, let $F_i:E_i^2\to[0,1]$ be the probability distribution defined by

$$\forall x,y\in E_i: F_i(x,y)=\frac{\pi(x)}{\pi(E_i)}\mathbb{P}_x(X_j=y|j<\tau_{\partial_i})=\pi_{|E_i}(x)\mathbb{P}_x(X_j=y|j<\tau_{\partial_i}). \tag{85}$$

Using this definition and recalling the definition of the Hellinger distance $d_H(p,q)=\sqrt{1-\langle p|q\rangle}$ for all probability distributions p and q,

$$\sum_{x,y\in E_i}\sqrt{\frac{\pi(x)\mathbb{P}_x(X_j=y|j<\tau_{\partial_i})\pi(y)\mathbb{P}_y(X_j=x|j<\tau_{\partial_i})}{\pi(E_i)^2}}=\langle F_i|F_i^T\rangle=1-d_H\left(F_i,F_i^T\right)^2. \tag{86}$$

By the triangle inequality,

$$\begin{aligned} d_H(F_i, F_i^T) &\leq d_H\left(F_i, \pi_{|E_i} \otimes \nu_i\right) + d_H\left(\pi_{|E_i} \otimes \nu_i, \nu_i \otimes \pi_{|E_i}\right) + d_H\left(\nu_i \otimes \pi_{|E_i}, F_i^T\right) \\ &= 2d_H\left(F_i, \pi_{|E_i} \otimes \nu_i\right) + d_H\left(\pi_{|E_i} \otimes \nu_i, \nu_i \otimes \pi_{|E_i}\right). \end{aligned} \tag{87}$$

Using that $(a+b)^2 \leq 2a^2 + 2b^2$ for all positive numbers a, b:

$$d_H\left(F_i, F_i^T\right)^2 \leq 8d_H\left(F_i, \pi_{|E_i} \otimes \nu_i\right)^2 + 2d_H\left(\pi_{|E_i} \otimes \nu_i, \nu_i \otimes \pi_{|E_i}\right)^2 = 1 - M_i. \tag{88}$$

Inserting this last bound in Equation 80 gives:

$$\langle \pi | D_j | \pi \rangle \geq \min_{1 \leq i \leq m} \min_{x \in E_i} \mathbb{P}_x(j < \tau_{\partial_i}) \sum_{i=1}^{m} \pi(E_i) M_i. \tag{89}$$

□

G. Geometric reversibilization

Under weak assumptions, there is an ergodic Markov kernel converging to the most reversible distribution whose spectrum is, up to a multiplicative constant, the spectrum of the flat discriminant. This kernel is called the geometric reversibilization of P and its mixing time governs the complexity of the quantum algorithm. Defining the geometric reversibilization requires to first introduce the concept of ν reversibilization.

Definition 19. Let $\nu : \mathbb{S} \to]0,1[$ be a full support probability distribution on $\mathbb{S}$ (i.e. such that $\nu(x) > 0$ for each $x \in \mathbb{S}$). Define the ν reversibilization P_ν of P by:

$$P_\nu(x,y) = \frac{\nu(y)}{\nu(x)} P(y,x) \tag{90}$$

for each $x, y \in \mathbb{S}$.

Note that $P_\pi = P^\star$ and that P_ν is not necessarily a Markov kernel. The following proposition states a few basic properties of the ν reversibilization.

Proposition 25. For any full support probability distribution, P_ν has the same eigenvalues as P. If P is irreducible, P_ν is also irreducible. P_ν is a Markov kernel if and only if P has ν for stationary distribution. However, in any case, $\nu P_\nu = \nu$.

Let us now recall the definition of a primitive matrix.

Definition 20. A real matrix A is primitive if there exists $m \in \mathbb{N}$ such that all the entries of A^m are strictly positive.

If the flat discriminant is primitive, the Perron-Frobenius Theorem 6 ensures that it has a strictly positive eigenvector associated to its largest eigenvalue. As a consequence, the μ reversibilization P_μ of P is well-defined.

Proposition 26. Assume that the flat discriminant D of P is primitive. Let μ be the leading eigenvector of D with eigenvalue $o \in]0,1]$. Define the following matrix $Q : \mathbb{S}^2 \to \mathbb{R}_+$ by:

$$\forall x, y \in \mathbb{S} : Q(x,y) = \frac{1}{o}\sqrt{P(x,y)P_\mu(x,y)}. \tag{91}$$

Then, Q is a μ-reversible and ergodic Markov kernel with spectral gap $\gamma(Q)$. Also, the second largest eigenvalue λ_1 of D satisfies:

$$\lambda_1 = o(1 - \gamma(Q)). \tag{92}$$

More generally, $\sigma(D) = o\sigma(Q)$.

Proof. Let us start by showing that Q is a Markov kernel. Let $x \in \mathbb{S}$. Since $|\mu\rangle$ is the leading eigenvector of D with eigenvalue o:

$$\sum_{y\in\mathbb{S}} Q(x,y) = \frac{1}{o}\sum_{y\in\mathbb{S}} \sqrt{P(x,y)P_\mu(x,y)} = \frac{1}{o}\sum_{y\in\mathbb{S}} \sqrt{\frac{\mu(y)}{\mu(x)}}D(x,y) = \frac{o\sqrt{\mu(x)}}{o\sqrt{\mu(x)}} = 1. \tag{93}$$

Note that $Q(x,y) > 0$ if and only if $D(x,y) > 0$. It follows that Q is ergodic if and only if D is primitive. Since we assume D is primitive, Q is ergodic. In fact, Q is reversible with respect to μ:

$$\forall x, y \in \mathbb{S} : \mu(x)Q(x,y) = \frac{1}{o}\sqrt{\mu(x)P(x,y)\mu(x)P_\mu(x,y)} = \frac{1}{o}\sqrt{\mu(y)P_\mu(y,x)\mu(y)P(y,x)} = \mu(y)Q(y,x). \tag{94}$$

The fact that $o\sigma(Q) = \sigma(D)$ comes from the fact that oQ is similar to D. □

Proposition 26 shows in particular that the following definition is well-posed as soon as the flat discriminant is primitive.

Definition 21. Let P be an ergodic Markov kernel with primitive flat discriminant D. Let $|\mu\rangle$ be the leading eigenvector of D with eigenvalue $o \in]0,1]$. Then, define the geometric reversibilization Q of P by:

$$Q(x,y) = \frac{1}{o}\sqrt{P(x,y)P_\mu(x,y)}. \tag{95}$$

Note that if P is reversible, then it is equal to its geometric reversibilization. This kernel is useful to locate the eigenvalues of the flat discriminant. If $\langle\pi|D|\pi\rangle$ and μ can be computed, as in the case of random walks on groups for example, then the geometric reversibilization can be implemented using the results from Section II.

V. EXAMPLES

This section illustrates the results from Section IV on concrete Markov chains using analytic and numerical methods.

A. Exponential speedup for the winning streak Markov chain

Note that, when a Markov chain is not reversible, it may not mix at the same rate as its time reverse (in total variation). The winning streak Markov chain is an example where this phenomenon occurs. Let us recall its definition. $(X_t)_{t\in\mathbb{N}}$ has state space $\mathbb{S} = \{0, ..., N\}$, for some integer $N \in \mathbb{N}$. Its non-zero transitions are given by:

$$\begin{cases} P(i,0) = 1/2, 0 \le i \le N, \\ P(i,i+1) = 1/2, 0 \le i < N, \\ P(N,N) = 1/2. \end{cases} \tag{96}$$

The winning streak Markov chain mixes in constant time whereas its adjoint mixes in a time proportional to its state space size [8]. However, recall from Proposition 12 that the flat discriminant is common to P and $P^\star$. It follows from Proposition 17 that, up to a logarithmic dependence in the system size n, D_j tends to $|\pi\rangle\langle\pi|$ faster than the smallest of the mixing times of P and $P^\star$. To be more precise, if $j \ge \tau(\epsilon)$ for $\epsilon < 1/|\mathbb{S}|$, the eigenvalues of D can be separated by a constant degree polynomial and $|\mu\rangle$ is such that $\langle\mu|\pi\rangle \ge 1 - \epsilon + \mathcal{O}(\epsilon^2)$. Supplementary Figure 1a illustrates the first eigenvalues of D_j as j increases and compares them with convergence properties of $(P^\star)^j$. The leading eigenvector of D_j has good overlap with $|\pi\rangle$ exponentially faster than the mixing time and can be prepared with constant overhead from this point.

B. Random walk on a group

Let $(\mathbb{S}, \circ) = (\mathbb{Z}/N\mathbb{Z}, +)$, for $N = 401$. Define the probability measure $\nu : \mathbb{S} \to [0,1]$ by $\nu(1) = 3/4$ and $\nu(-1) = 1/4$. Let P_0 be the random walk on $(\mathbb{S}, \circ)$ associated with the measure ν (as in Definition 17). Let $P = P_0^{6N}$. Supplementary Figure 1b displays the spectra of P, its additive reversibilization $P_A = \frac{P+P^\star}{2}$ and its flat discriminant D. Proposition 18 ensures that to each eigenvalue of P, there is an eigenvalue of D within distance $c\sqrt{1 - \langle\pi|D|\pi\rangle}$. This property

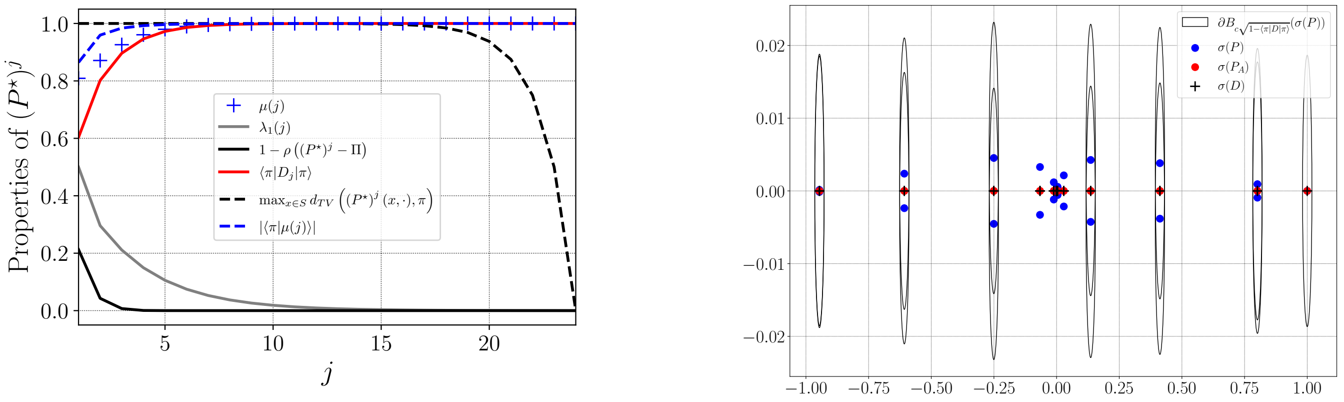

(a) Exponential speedup for the time-reversed winning streak Markov chain with $N = 24$. D_j denotes the flat discriminant of $(P^\star)^j$. $\mu(j)$ and $\lambda_1(j)$ denote its largest and second largest eigenvalues. $1 - \rho\left((P^\star)^j - \Pi\right)$ denotes the absolute spectral gap of $(P^\star)^j$.

(b) Spectral properties of $P, P_A = (P + P^\star)/2$ and D for a random walk on a group. The circles centered on the leading eigenvalues of P contain eigenvalues of D, as ensured by Proposition 18. As stated in Proposition 22, the eigenvalues of D are $1 - \langle\pi|D|\pi\rangle$ close to those of P_A.

Supplementary Figure 1

is illustrated by the circles centered on the leading eigenvalues of P. Proposition 22 uses the group property of the Markov chain to give a tighter bound. It says that to each eigenvalue of P_A, there is an eigenvalue of D within distance $1 - \langle\pi|D|\pi\rangle$. This property can be observed by comparing the positions of the eigenvalues of P_A and those of D. The reflection through $|\pi\rangle$ can be achieved with $\mathcal{O}(\sqrt{N})$ uses of the quantum walk operator associated with P whereas the mixing time of P is in $\mathcal{O}(N)$.

C. Markov chain on a graph with bottleneck

In order to illustrate the content of Proposition 23, let us give an example of a Markov chain with fast convergence to quasi-stationary distributions. Let N be odd ($N = 31$ for the numerical experiment) and consider the state space $\mathbb{S} = \{0, ..., N-1\} \cup \{\partial\} \cup \{N, ..., 2N-1\}$. Define the nonreversible Markov chain P by:

$$\begin{aligned} \forall x \in \{1, ..., N-1\} : P(x, (x+1)[N]) = P(N+x, N+(x+1)[N]) = 3/4, \\ \forall x \in \{1, ..., N-1\} : P(x, (x-1)[N]) = P(N+x, N+(x-1)[N]) = 1/4, \end{aligned} \tag{97}$$

where $[N]$ denotes modulo N and

$$\begin{cases} P(0,\partial) = P(N,\partial) = 1/N^3, \\ P(0,1) = P(N,N+1) = (1-1/N^3)3/4, \\ P(0,N-1) = P(N,2N-1) = (1-1/N^3)/4 \\ P(\partial,0) = P(\partial,N) = 1/2. \end{cases} \tag{98}$$

If the process starts in $\{0, N-1\}$ and does not exit this set, then it converges to a quasi-stationary distribution in $\mathcal{O}(N^2)$ steps. The same holds if the starting set is $\{N, 2N-1\}$. Since it takes of the order of N^3 steps to exit the set, the overall mixing time is at least of order N^3. Supplementary Figure 2 shows that the fast convergence to a quasi-stationary is sufficient for D to present the desired spectral properties.

D. Biased random walk and its geometric reversibilization

Let $p \in]0,1[$ be a fixed parameter. Let $\mathbb{S}_N = \mathbb{Z}/N\mathbb{Z}$. Consider the Markov kernel P_p on S_N that moves right with probability p and left with probability $1-p$. The chain is known to mix in time $\mathcal{O}(N^2)$. On the other hand, the flat discriminant of the chain is $D_p = \langle\pi|D_p|\pi\rangle\, P_{1/2} = 2\sqrt{p(1-p)}P_{1/2}$. The associated geometric reversibilization is therefore $Q_p = P_{1/2} = (P_p + P_p^\star)/2$ for any $p \in]0,1[$. Its associated quantum walk operator can easily be implemented

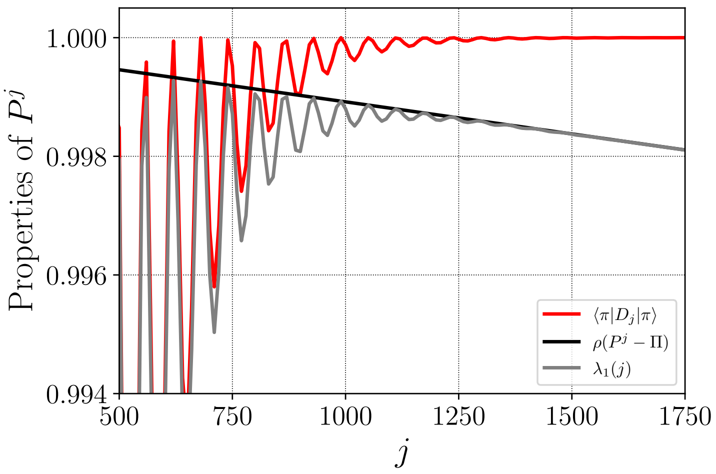

Supplementary Figure 2: Comparison between $\langle\pi|D_j|\pi\rangle$, the second largest eigenvalue of D_j and the absolute spectral gap of P^j for a Markov chain with two components connected by a bottleneck. The time required for convergence to a quasi-stationary distribution is much smaller than the mixing time and sufficient for the leading eigenvalue of D_j to be close to 1 and the second largest to be close to the spectral radius of $P^j - \Pi$.

to yield a total reflection construction time in $\mathcal{O}(N)$. This procedure is as efficient as the direct transformation of the singular values of the curved discriminant $\mathcal{D}_p$ of P_p since P_p is normal. Also, note that according to Proposition 26, $\sigma(D_p) = \langle\pi|D_p|\pi\rangle\, \sigma\left((P_p + P_p^\star)/2\right)$ for any $p \in]0,1[$. This result is more precise than the one given by Proposition 22 which says that the eigenvalues of D_p and of $(P_p + P_p^\star)/2$ are $1 - \langle\pi|D_p|\pi\rangle$ close.

E. Nonreversible walk on the circle

1. Intuition

Let us consider the walk on the circle, with speed-reversal probability 1/2. The Markov chain $(X_n, V_n)_{n\in\mathbb{N}}$ takes values in $\mathbb{S} = \mathbb{Z}/N\mathbb{Z} \times \{1, -1\}$ for some odd integer $N \in 2\mathbb{Z}+1$. It evolves according to:

$$(X_{n+1}, V_{n+1}) = \begin{cases} (X_n + V_n, V_n) \text{ with probability } 1/2, \\ (X_n, -V_n) \text{ with probability } 1/2. \end{cases} \tag{99}$$

Its transition matrix being doubly stochastic, it converges to the uniform distribution. If the circle is large enough, the chain rapidly approaches a Gaussian distribution (in space) about the starting point (with probability 1/2 of being in each speed). As a consequence, the overlap $\langle P(x,\cdot)|P^\star(x,\cdot)\rangle$ approaches 1 much faster than the mixing time for each $x \in \mathbb{S}$. Section V E 2 gives a rigorous upper bounds on the second largest eigenvalue of D_j in terms of $\langle\pi|D_j|\pi\rangle$. Section V E 3 shows that $\langle\pi|D_j|\pi\rangle \to 1$ at rate $1/j$, which is of the order of the square root of the mixing time of the chain. Supplementary Figure 3a shows that the eigenvalues of D_j remain within distance $1 - \langle\pi|D_j|\pi\rangle$ of an eigenvalue of P. Supplementary Figure 3b shows that $\langle\pi|D_j|\pi\rangle$ tends quadratically faster to 1 than $\mathbb{E}_\pi\left[d_{TV}(P^j(x,\cdot), (P^\star)^j(x,\cdot))\right]$ tends to 0.

2. Second largest eigenvalue of the discriminant

Note that the spectral radius of $\mathcal{D}_A - D$ is upper bounded by the sum of its lines. In particular, when π is uniform and the L^1 norm of the lines is constant:

$$\forall x \in \mathbb{S} : \frac{1}{2}\sum_y P(x,y) + P(y,x) - 2\sqrt{P(x,y)P(y,x)} = 1 - \langle\pi|D|\pi\rangle\,. \tag{100}$$

Let us maximize $\langle f, Df\rangle$ with the constraint that f must be orthogonal to the leading eigenvector of D. For this

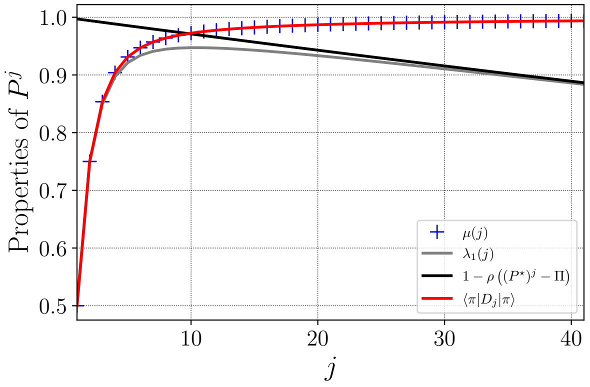

(a) Kinetic walk on the circle. $N = 41$. Properties of $D_j(x, y) = \sqrt{P^j(x, y)P^j(y, x)}$ as j increases.

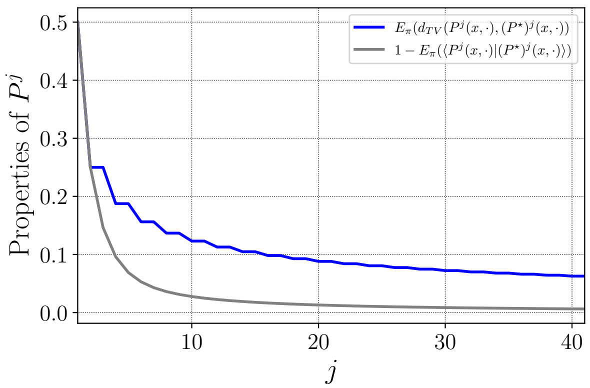

(b) $1 - \mathbb{E}_\pi\left[\langle P(x,\cdot)|P^\star(x,\cdot)\rangle\right]$ behaves like the square of $\mathbb{E}_\pi\left[d_{TV}(P(x,\cdot), P^\star(x,\cdot))\right]$ for the simple kinetic walk.

Supplementary Figure 3

simple example, the leading eigenvector of D is precisely $|\pi\rangle$. Therefore,

$$|\langle f, Df\rangle| \leq (1 - \langle\pi|D|\pi\rangle)\langle f, f\rangle + (1 - \gamma(P))\langle f, f\rangle\,, \tag{101}$$

where $\gamma(P)$ is the Poincaré constant of P. It follows that:

$$\forall\lambda \in \sigma(D)\backslash\{1\} : |\lambda| \leq 1 - \gamma(P) + 1 - \langle\pi|D|\pi\rangle\,. \tag{102}$$

It is known that $\gamma(P)$ behaves as the absolute spectral gap and as the inverse of the mixing time for this problem [10].

3. *Scaling of* $\langle\pi|D_j|\pi\rangle$

Proposition 27. Let $0 < y < N$ and $1 \leq j < N$. Then,

$$\begin{cases} D_j((0,+),(y,+)) = \dfrac{1}{2^j}\sqrt{\dbinom{j-1}{\frac{j+y-2}{2}}\dbinom{j-1}{\frac{j+y}{2}}}, \\ D_j((0,+),(y,-)) = \dfrac{1}{2^j}\dbinom{j-1}{\frac{j-1+y}{2}}, \end{cases} \tag{103}$$

where $\dbinom{a}{b} = 0$ by convention if $b \notin \mathbb{N}$.

Let us recall the following facts about the simple random walk on $\mathbb{Z}$.

Lemma 4. Let $a \leq b \in \mathbb{N}$ and $(W_n)_{n\in\mathbb{N}}$ be a simple random walk on $\mathbb{Z}$ starting at $W_0 = 0$. Then,

$$\mathbb{P}_0(W_b = a) = \frac{1}{2^b}\binom{b}{\frac{a+b}{2}}. \tag{104}$$

Proof. Let us introduce a sequence of random variables $(S_n)_{n\in\mathbb{N}}$ by $S_0 = 1/2$ and $S_{n+1} = S_n + V_{n+1}$ (where (X_n, V_n) is the Markov chain following P with intial condition $(X_0, V_0) = (0,+)$). Note that for all $n \in \mathbb{N}$, if $V_n = +$, then $X_n = S_n - 1/2$ and if $V_n = -$, then $X_n = S_n + 1/2$. Note that by symmetry:

$$D_j((0,+),(y,-)) = P^j((0,+),(y,-)). \tag{105}$$

Moreover,

$$\begin{aligned} P^j((0,+),(y,-)) &= \mathbb{P}_{(0,+)}((X_j,V_j)=(y,-)) \\ &= \mathbb{P}_{(0,+)}(S_j+1/2,V_j)=(y,-)) \\ &= \mathbb{P}_{(0,+)}(\{S_j+1/2=y\}\cap\{S_j=S_{j-1}-1\}) \\ &= \mathbb{P}_{(0,+)}(\{S_{j-1}-1/2=y\}\cap\{S_j=S_{j-1}-1\}) \\ &= \frac{1}{2^j}\binom{j-1}{\frac{j-1+y}{2}} \end{aligned} \tag{106}$$

by Lemma 4. A similar reasoning yields

$$\begin{aligned} P^j((0,+),(y,+)) &= \mathbb{P}_{(0,+)}((X_j,V_j)=(y,+)) \\ &= \mathbb{P}_{(0,+)}(S_j-1/2,V_j)=(y,+)) \\ &= \mathbb{P}_{(0,+)}(\{S_j-1/2=y\}\cap\{S_j=S_{j-1}+1\}) \\ &= \mathbb{P}_{(0,+)}(\{S_{j-1}-1/2=y-1\}\cap\{S_j=S_{j-1}+1\}) \\ &= \frac{1}{2^j}\binom{j-1}{\frac{j+y-2}{2}} \end{aligned} \tag{107}$$

and

$$\begin{aligned} P^j((0,+),(-y,+)) &= \mathbb{P}_{(0,+)}((X_j,V_j)=(-y,+)) \\ &= \mathbb{P}_{(0,+)}(S_j-1/2,V_j)=(-y,+)) \\ &= \mathbb{P}_{(0,+)}(\{S_j-1/2=-y\}\cap\{S_j=S_{j-1}+1\}) \\ &= \mathbb{P}_{(0,+)}(\{S_{j-1}-1/2=y+1\}\cap\{S_j=S_{j-1}+1\}) \\ &= \frac{1}{2^j}\binom{j-1}{\frac{j+y}{2}}. \end{aligned} \tag{108}$$

Using that $P^j((y,+),(0,+)) = P^j((0,+),(-y,+))$,

$$D_j((0,+),(y,+)) = \frac{1}{2^j}\sqrt{\binom{j-1}{\frac{j+y-2}{2}}\binom{j-1}{\frac{j+y}{2}}}. \tag{109}$$

□

Proposition 28. As $j,N\to\infty$ with $j<N$:

$$\langle\pi|D_j|\pi\rangle = 1-\mathcal{O}\left(\frac{\ln(j)}{j}\right). \tag{110}$$

Recall the following form of Stirling's approximation.

Lemma 5. Let $t\in\mathbb{N}$. As $t\to\infty$:

$$\ln(t!) = t\ln(t) - t + \frac{\ln(2\pi t)}{2} + \frac{1}{12t} + \mathcal{O}\left(\frac{1}{t^3}\right). \tag{111}$$

Proof. Recalling from Proposition 27, write for $0\le y\le j$:

$$D_j((0,+),(y,+)) = \frac{1}{2^j}\sqrt{\binom{j-1}{\frac{j+y-2}{2}}\binom{j-1}{\frac{j+y}{2}}} \tag{112}$$

In order to estimate the square root in the above equation, let us estimate the binomial coefficients $\binom{t}{\frac{t+m}{2}}$ for

$m \in \mathbb{N}, t \in \mathbb{N}\backslash\{0\}$. Using Lemma 5 and $\ln(1+x) = x - x^2/2 + x^3/3 + \mathcal{O}(x^4)$ as $x \to 0$:

$$\begin{aligned}\ln\left(\left(\frac{t+m}{2}\right)!\right) &= \frac{t+m}{2}\left(\ln(t) + \ln(1+m/t) - \ln(2) - 1\right) \\ &+ \frac{\ln(\pi)}{2} + \frac{\ln(t)}{2} + \frac{\ln(1+m/t)}{2} + \frac{1}{6(t+m)} + \mathcal{O}\left(\frac{1}{t^3}\right) \\ &= \frac{t+m}{2}\left(\ln(t) + \frac{m}{t} - \frac{m^2}{2t^2} + \frac{m^3}{3t^3} - \ln(2) - 1\right) \\ &+ \frac{\ln(\pi)}{2} + \frac{\ln(t)}{2} + \frac{m}{2t} - \frac{m^2}{4t^2} + \frac{1}{6t} - \frac{m}{6t^2} + \mathcal{O}\left(\frac{1}{t^3}\right).\end{aligned} \tag{113}$$

It follows that:

$$\begin{aligned}\ln\left(\left(\frac{t+m}{2}\right)!\left(\frac{t-m}{2}\right)!\right) &= t\left(\ln(t) - \frac{m^2}{2t^2} - \ln(2) - 1\right) + \frac{m^2}{t} \\ &+ \ln(\pi) + \ln(t) - \frac{m^2}{2t^2} + \frac{1}{3t} + \mathcal{O}\left(\frac{1}{t^3}\right) \\ &= t\left(\ln(t) - \ln(2) - 1\right) + \frac{m^2}{2t} \\ &+ \ln(\pi) + \ln(t) - \frac{m^2}{2t^2} + \frac{1}{3t} + \mathcal{O}\left(\frac{1}{t^3}\right).\end{aligned} \tag{114}$$

It is now possible to compute $\ln\binom{t}{\frac{t+m}{2}} = \ln(n!) - \ln\left(\left(\frac{t+m}{2}\right)!\left(\frac{t-m}{2}\right)!\right)$:

$$\ln\binom{t}{\frac{t+m}{2}} = t\ln(2) - \frac{m^2}{2t} + \frac{\ln(2/\pi)}{2} - \frac{\ln(t)}{2} + \frac{m^2}{2t^2} - \frac{1}{4t} + \mathcal{O}\left(\frac{1}{t^3}\right). \tag{115}$$

from which we can deduce:

$$\ln\left(\frac{1}{2^t}\binom{t}{\frac{t+m}{2}}\right) = -\frac{m^2}{2t} + \frac{\ln(2/\pi)}{2} - \frac{\ln(t)}{2} + \frac{m^2}{2t^2} - \frac{1}{4t} + \mathcal{O}\left(\frac{1}{t^3}\right). \tag{116}$$

Using this expression for each of the binomial coefficients, we obtain:

$$\begin{aligned}\ln\left(\frac{1}{2^t}\sqrt{\binom{t}{\frac{t+m-1}{2}}\binom{t}{\frac{t+m+1}{2}}}\right) &= -\frac{(m-1)^2+(m+1)^2}{4t} + \frac{\ln(2/\pi)}{2} - \frac{\ln(t)}{2} + \frac{(m-1)^2+(m+1)^2}{4t^2} - \frac{1}{4t} + \mathcal{O}\left(\frac{1}{t^3}\right) \\ &= -\frac{m^2+1}{2t} + \frac{\ln(2/\pi)}{2} - \frac{\ln(t)}{2} + \frac{m^2+1}{2t^2} - \frac{1}{4t} + \mathcal{O}\left(\frac{1}{t^3}\right).\end{aligned} \tag{117}$$

By comparison,

$$\begin{aligned}\ln\left(\frac{1}{2^{t+1}}\binom{t+1}{\frac{t+m+1}{2}}\right) &= -\frac{m^2}{2t} + \frac{m^2}{2t^2} + \frac{\ln(2/\pi)}{2} - \frac{\ln(t)}{2} - \frac{1}{2t} + \frac{1}{4t^2} - \frac{1}{4t} + \frac{1}{4t^2} + \frac{m^2}{2t^2} + \mathcal{O}\left(\frac{1}{t^3}\right) \\ &= -\frac{m^2+1}{2t} + \frac{\ln(2/\pi)}{2} - \frac{\ln(t)}{2} + \frac{m^2+1}{2t^2} - \frac{1}{4t} + \frac{m^2}{2t^2} + \mathcal{O}\left(\frac{1}{t^3}\right).\end{aligned} \tag{118}$$

Let $d \in \mathbb{N}$ be the smallest integer such that $e^{-\frac{d^2}{2t}} \leq 1/t$. It can be seen that $d \in \mathcal{O}(\sqrt{t\ln(t)})$. Then, it is a well-known consequence of Chebyshev's inequality that $1 - \mathbb{P}(|W_t| < d) \in \mathcal{O}(1/t)$. Also, if $-d < m < d$, then:

$$\ln\left(\frac{1}{2^{t+1}}\binom{t+1}{\frac{t+m+1}{2}}\right) \leq \ln\left(\frac{1}{2^t}\sqrt{\binom{t}{\frac{t+m-1}{2}}\binom{t}{\frac{t+m+1}{2}}}\right) + \frac{d^2}{2t^2} + \mathcal{O}\left(\frac{1}{t^3}\right). \tag{119}$$

so that:

$$\begin{aligned}\langle\pi|D_j|\pi\rangle &\geq \frac{1}{2}\sum_{m=-d}^{d}\frac{1}{2^{j-1}}\sqrt{\binom{j-1}{\frac{j+m}{2}}\binom{j-1}{\frac{j+2+m}{2}}}+\frac{1}{2}\\ &\geq \frac{1}{2}\sum_{m=-d}^{d}e^{-\frac{d^2}{2j^2}+\mathcal{O}(1/j^3)}\frac{1}{2^j}\binom{j}{\frac{j+m}{2}}+\frac{1}{2}\\ &\geq 1-\mathcal{O}\left(\frac{\ln(j)}{j}\right).\end{aligned} \tag{120}$$

□

As a consequence, $\mathcal{O}\left(1/\sqrt{N}\right)$ calls to the projected unitary encoding of D_N allow to reflect through the stationary distribution. A naive implementation of this projected unitary encoding has cost $\mathcal{O}(N)$, resulting in a total cost of $\mathcal{O}\left(N^{3/2}\right)$. This is already smaller than the mixing time of the chain in $\mathcal{O}\left(N^2\right)$.

Proposition 29. As $j, N \to \infty$ with $j < N$:

$$\mathbb{E}_\pi\left[d_{TV}(P^j(x,\cdot),(P^\star)^j(x,\cdot))\right] \in \mathcal{O}\left(\frac{1}{\sqrt{j}}\right). \tag{121}$$

Proposition 29 will be proved using the concept of Markovian coupling [8].

Definition 22. Given a Markov kernel P with finite state space $\mathbb{S}$, a Markovian coupling of two chains $(X_t)_{t\in\mathbb{N}}$ and $(Y_t)_{t\in\mathbb{N}}$ with respective initial conditions $X_0, Y_0 \in \mathbb{S}$ is a Markov chain $(X_t, Y_t)_{t\in\mathbb{N}}$ on $\mathbb{S}^2$ which satisfies:

$$\forall x,y,x',y' \in \mathbb{S}: \begin{cases}\mathbb{P}(X_{t+1}=x'|X_t=x,Y_t=y)=P(x,x')\\ \mathbb{P}(Y_{t+1}=y'|X_t=x,Y_t=y)=P(y,y').\end{cases} \tag{122}$$

Proof. We have that

$$\mathbb{E}_\pi\left[d_{TV}(P^j(x,\cdot),(P^\star)^j(x,\cdot))\right] = \frac{1}{2}d_{TV}(\mu_j,\nu_j), \tag{123}$$

where $\mu_j(x) = \mathbb{P}(W_{j-1} = x-1|W_0 = 0)$ and $\nu_j(x) = \mathbb{P}(W_{j-1} = x+1|W_0 = 0) = d_{TV}(Q^{j-1}(1,\cdot),Q^{j-1}(-1,\cdot))$, where $(W_m)_{m\in\mathbb{N}}$ is a simple random walk on $\mathbb{Z}$ and $x \in \mathbb{Z}$. More precisely, the stochastic process $(W_m)_{m\in\mathbb{N}}$ follows the Markov kernel $Q(x,x+1) = Q(x,x-1) = 1/2$ for all $x \in \mathbb{Z}$. Define a Markovian coupling starting in position $(1,-1)$ such that before the chains touch, they move in opposite directions, and once they touch, they remain equal. According to Theorem 5.4 in [8], $d_{TV}(\mu_j,\nu_j) \leq \mathbb{P}_{1,-1}(\tau_0 \geq j)$. Here, τ_0 is the coalescence time of the chains, defined by $\tau_0 = \min(t \in \mathbb{N} : \forall s \geq t : X_s = Y_s)$. Conclude using Proposition 17.19 in [8] that $\mathbb{P}(\tau_0 \geq j) \leq 2/\sqrt{j}$. □

Proposition 30. As $j, N \to \infty$ with $j < N$:

$$\mathbb{E}_\pi\left[d_{TV}(P^j(x,\cdot),(P^\star)^j(x,\cdot))\right] \in \Omega\left(\frac{1}{\sqrt{j}}\right). \tag{124}$$

Proof. Define μ_j and ν_j as in the proof of Proposition 29. For all subsets of A of $\mathbb{Z}$, $d_{TV}(\mu_j,\nu_j) \geq \mu_j(A) - \nu_j(A)$. Let $A = \{n \in \mathbb{Z} : n \geq 1\}$. Then, $\nu_j(A) = \mu_j(A) - \mathbb{P}(Z_{j-1} = 0) - \mathbb{P}(Z_{j-1} = -1)$. By Lemma 5:

$$\mathbb{E}_\pi\left[d_{TV}(P^j(x,\cdot),(P^\star)^j(x,\cdot))\right] \geq \frac{1}{2}\sqrt{\frac{2}{\pi j}}e^{\mathcal{O}(1/j)}. \tag{125}$$

□

In fact, combining the proofs of Propositions 29 and 30 yields the following result.

Corollary 7. As $j, N \to \infty$ with $j < N$:

$$\mathbb{E}_\pi\left[d_{TV}(P^j(x,\cdot),(P^\star)^j(x,\cdot))\right] \in \Theta\left(\frac{1}{\sqrt{j}}\right). \tag{126}$$

4. Geometric reversibilization

For this example, the most reversible distribution is known, and $\langle\pi|D_2|\pi\rangle$ can be computed. As a consequence, the geometric reversibilization Q_2 of P^2 can be implemented using the methods from Section II. Moreover, Q_2 has a spectral gap in $\mathcal{O}\left(1/N^2\right)$. The reflection through the stationary distribution can be implemented with $\mathcal{O}(N)$ uses of the quantum walk operators associated with $(1+Q_2)/2$. In particular, it is possible to use properties of the geometric reversibilization to improve the method relying only on the flat discriminant.

5. Example of quantum circuit

The Szegedy Quantum Walk reflections R are typically implemented using oracles $\mathcal{P}$ that perform:

$$\mathcal{P}\,|x\rangle\,|0\rangle = |\psi_x\rangle\,. \tag{127}$$

Indeed, this is sufficient since $R = 2\sum_{x\in\mathbb{S}}|\psi_x\rangle\langle\psi_x| - 1 = \mathcal{P}\left(2\sum_{x\in\mathbb{S}}|x\rangle\,|0\rangle\,\langle x|\,\langle 0| - 1\right)\mathcal{P}^\dagger = \mathcal{P}(1\otimes(2\,|0\rangle\,\langle 0| - 1))\mathcal{P}^\dagger$ and $2\,|0\rangle\,\langle 0| - 1$ can easily be implemented. It suffices to set a clean ancilla to $|1\rangle$ if and only if the qubits are in (multi-qubit) state $|0\rangle$, applying a Z gate on the ancilla and restoring the ancilla to $|0\rangle$ [11, 12]. $\mathcal{P}$ is most often constructed with rather costly reversible arithmetic oracles [11]. However, it is often possible to find simpler and more efficient construction [13]. Supplementary Figure 4 gives an example construction for the kinetic walk in a constant potential in the case $N = 2^n - 1$, for some $n \geq 1$. The single-qubit registers v_1 and v_2 store the velocity of each of the copies of the state space. The n-qubit registers x_1 and x_2 store the position of each of the copies of the state space. For $0 \leq \alpha \leq 1$, $R(\alpha)$ is the single-qubit operator that flips the velocity with probability $(1-\alpha)/2$:

$$R(\alpha) = \begin{pmatrix} \sqrt{\frac{1+\alpha}{2}} & \sqrt{\frac{1-\alpha}{2}} \\ \sqrt{\frac{1-\alpha}{2}} & \sqrt{\frac{1+\alpha}{2}} \end{pmatrix}. \tag{128}$$

Σ is the increment operator, such that $\Sigma\,|x\rangle = |x+1\rangle$ for each $x \in \mathbb{Z}/(N+1)\mathbb{Z}$. Also, $\mathcal{X} = \sum_{x=1}^{N-1}|x\rangle\,\langle x| + |N\rangle\,\langle 0| + |0\rangle\,\langle N|$ such that $X'\Sigma = \sum_{x=0}^{N-1}|(x+1)[N]\rangle\,\langle x| + |N\rangle\,\langle N|$. $\mathcal{X}$ is straightforward to implement using a Grey code [11].

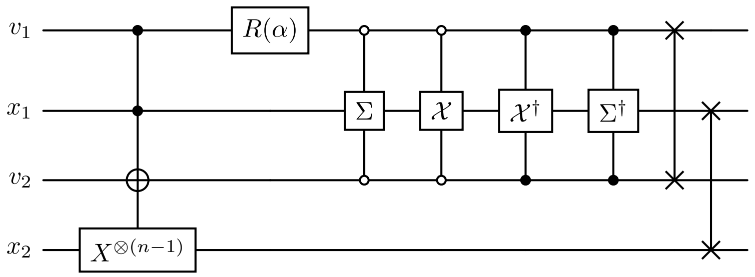

Supplementary Figure 4: Circuit for the Szegedy Quantum Walk operator of the kinetic walk.

F. Kinetic walks for sampling

Let us introduce a nonreversible kinetic Markov chain [14] on state space $\mathbb{S}_N = \mathbb{Z}/N\mathbb{Z}\times\{1,-1\}$, for some $N \in 2\mathbb{N}+1$, with chosen velocity-independent stationary distribution $\pi(x,v) = \frac{\pi(x)}{2}$ for each $(x,v) \in \mathbb{S}_N$. The Markov kernel is defined as $P = QR$ with:

$$\begin{cases} Q((x,v),(x+v,-v)) = \min\left(1, \dfrac{\pi(x+v)}{\pi(x)}\right), \\ Q((x,v),(x,v)) = 1 - \min\left(1, \dfrac{\pi(x+v)}{\pi(x)}\right), \end{cases} \tag{129}$$

and

$$\begin{cases} R((x,v),(x,-v)) = 1 - 1/N, \\ R((x,v),(x,v)) = 1/N \end{cases} \tag{130}$$

for each $(x,v) \in \mathbb{S}_N$. For the rest of this section, assume that for each $x \in \mathbb{Z}/N\mathbb{Z}$, $\pi(x,v) = \exp(-U(x/N))/\mathcal{Z}$ for a $C^2(\mathbb{R})$ and 1-periodic function U and $\mathcal{Z}$ a normalization constant such that π is a valid probability distribution on $\mathbb{S}_N$. We will show that P^N is a discrete approximation of a nonreversible Markov process that can be efficiently simulated using the quantum algorithms introduced in the previous sections.

Lemma 6. There exists constants $l, l' > 0$ and $n_0 \in \mathbb{N}$ such that for all $N \geq n_0$:

$$\forall (x,v) \in \mathbb{S}_N : P^N((x,v),(x,v)) \geq l \tag{131}$$

and

$$\forall (x,v) \in \mathbb{S}_N : P^{2N}((x,v),(x,v)) \geq l'. \tag{132}$$

Proof. Let $(x_N, v) \in \mathbb{S}$ be such that $x_N/N = \tilde{x} \in [0,1]$ for each $N \in \mathbb{N}$. Since the probability of returning to its initial position and speed after N steps is greater than the probability of doing a full loop around the circle:

$$\begin{aligned} P^N((x_N,v),(x_N,v)) &\geq \left(1-\frac{1}{N}\right)^N \prod_{k=1}^{N} \exp\left(-\left[U\left(\frac{x_N+kv}{N}\right) - U\left(\frac{x_N+(k-1)v}{N}\right)\right]_+\right) \\ &= \left(1-\frac{1}{N}\right)^N \exp\left(-\frac{2}{N}\sum_{k=1}^{N} \frac{\left[U\left(\tilde{x}+\frac{kv}{N}\right) - U\left(\tilde{x}+\frac{(k-1)v}{N}\right)\right]_+}{2/N}\right) \\ &\to e^{-1}\exp\left(-2\int_0^1 [vU'(vs)]_+ ds\right) > 0. \end{aligned} \tag{133}$$

Conclude by noting that $P^{2N}((x_N,v),(x_N,v)) \geq P^N((x_N,v),(x_N,v))^2$. □

Lemma 7. There is a constant $C > 0$ such that for all $N \in \mathbb{N}$:

$$\forall (x,v) \in \mathbb{S}_N : P((x,v),(x,-v)) \geq \frac{C}{N}. \tag{134}$$

Proof. Let $(x,v) \in \mathbb{S}_N$. Then,

$$\begin{aligned} P((x,v),(x,-v)) &= Q((x,v),(x,-v))R((x,-v),(x,-v)) + Q((x,v),(x,v))R((x,v),(x,-v)) \\ &\geq Q((x,v),(x,-v))R((x,-v),(x,-v)) \\ &= \frac{1}{N}\exp\left(-\left[U\left(\frac{x+v}{N}\right) - U\left(\frac{x}{N}\right)\right]_+\right) \\ &\geq \frac{1}{N}e^{-\|U'\|_\infty/N}. \end{aligned} \tag{135}$$

□

Lemma 8. There exists $l > 0$ and $n_0 \in \mathbb{N}$ such that for all $x, y, k \in \mathbb{Z}/N\mathbb{Z}$, $v \in \{1,-1\}$ and $N \geq n_0$:

$$P^k((x,v),(x+kv,v)) > l \tag{136}$$

and

$$P^{N-1-k}((x+kv,-v),(y,-v)) > l. \tag{137}$$

Proof. That is a simple corollary from Lemma 6. Indeed for each $1 \leq k < N$, $P^k((x,v),(x+kv,v))$ is greater than the probability of doing a full loop around the circle. As shown in Lemma 6, this probability converges to a strictly positive number. The first property is trivial when $k = 0$ and therefore proven. The second property can be proven the same way. □

Proposition 31. P^{2N} satisfies a Doeblin condition with constant independent from N.

Proof. Our goal is to show that there exists a constant $c > 0$ such that $P^{2N}((x,v),(y,w)) \geq c/N$ for each $(x,v),(y,w) \in \mathbb{S}$. We will treat the cases where $x-y$ is even or odd separately. Recall that for $v \in \{-1,+1\}$ and $x \in \mathbb{Z}/N\mathbb{Z}$, $P^N((x,v),(x,v))$ and $P^{2N}((x,v),(x,v))$ are bounded from below by a constant (as stated in Lemma 6).

- Let $y \neq x \in \mathbb{Z}/N\mathbb{Z}$ be such that x and y have different parity. Let us prove that $P^{2N}((x,v),(y,-v)) \in \Omega(1/N)$.

Write $y - x = 2q + v$ for some $q \in \mathbb{Z}$. Write $q = vk[N]$, for some $0 \leq k < N$. Then,

$$y = x + 2q + v = x + kv - v(N-1-k)[N]. \tag{138}$$

Therefore, using Lemma 7 and Lemma 8,

$$P^N((x,v),(y,-v)) \geq P^k((x,v),(x+kv,v))P((x+kv,v),(x+kv,-v))P^{N-1-k}((x+kv,-v),(y,-v)) \in \Omega(1/N). \tag{139}$$

It follows that $P^{2N}((x,v),(y,-v)) \geq P^N((x,v),(y,-v))P^N((y,-v),(y,-v)) \in \Omega(1/N)$.

- Let $y \neq x \in \mathbb{Z}/N\mathbb{Z}$ be such that x and y have different parity. Let us prove that $P^{2N}((x,v),(y,v)) \in \Omega(1/N)$.

Since $x \neq y$, the chain must change direction at least twice to go from x to y in N steps while finishing at its initial speed. Assume that the chain performs $a \in \mathbb{N}$ steps with its initial speed, changes direction, $k \in \mathbb{N}$ steps with the opposite speed, changes direction and $b \in \mathbb{N}$ steps with its initial speed. Let $y - x = d[N]$, with $0 \leq d < N$. a, k and b must satisfy the following set of equations:

$$\begin{cases} a - k + b = d \\ a + k + b = N - 2 \end{cases} \tag{140}$$

In particular, $b(a) \equiv \frac{d+N-2}{2} - a$ and $k(a) = \frac{N-2-d}{2}$ are such that $a, b(a)$ and $k(a)$ are solutions, as long as $0 \leq a \leq \frac{d+N-2}{2}$. The probability of one such trajectory is:

$$\begin{aligned} &P^a((x,v),(x+va,v))P((x+va,v),(x+va,-v))P^{k(a)}((x+va,-v),(x+va-k(a)v,-v)) \\ &\times P((x+va-k(a)v,-v),(x+va-k(a)v,v))P^{b(a)}((x+va-k(a)v,v),(y,v)) \\ &\in \Omega\left(\frac{1}{N^2}\right). \end{aligned} \tag{141}$$

Since there are $\Theta(N)$ such trajectories, $P^N((x,v),(y,v)) \in \Omega(1/N)$. Conclude as before using $P^{2N}((x,v),(y,v)) \geq P^N((x,v),(y,v))P^N((y,v),(y,v))$.

- Let $y \neq x \in \mathbb{Z}/N\mathbb{Z}$ be such that x and y have the same parity. Let us prove that $P^{2N}((x,v),(y,v)) \in \Omega(1/N)$.

Indeed,

$$\begin{aligned} P^{2N}((x,v),(y,v)) &\geq \sum_{z \in \mathbb{Z}/N\mathbb{Z}} P^N((x,v),(z,-v))P^N((z,-v),(y,v)) \\ &\in \Omega(1/N), \end{aligned} \tag{142}$$

since we can restrict the sum to z of opposite parity to both x and y and use Equation 139.

- Let $y \neq x \in \mathbb{Z}/N\mathbb{Z}$ be such that x and y have the same parity. Let us prove that $P^{2N}((x,v),(y,-v)) \in \Omega(1/N)$.

Note that

$$P^{2N}((x,v),(y,-v)) \geq \sum_z P^N((x,v),(z,-v))P^N((z,-v),(y,-v)) \in \Omega(1/N) \tag{143}$$

since we can restrict the sum to z of opposite parity to both x and y and use Equation 141.

Proposition 36 allows to conclude. □

Note that once P^{2N} satisfies a Doeblin condition with constant α independent from N, $(P^\star)^{2N}P^{2N}$ satisfies a Doeblin condition with constant $(1-\alpha)^2 = 1 - 2\alpha + \mathcal{O}(\alpha^2)$. If α is small, it is interesting to use the quantum algorithm with Markov kernel P^N, even though P^N is not reversible.

G. Out-of-equilibrium kinetic walk

The out-of-equilibirum kinetic walk kernel is a linear combination from the previous walk and a modified version which always goes in one direction. Let Q be a Markov kernel that suggests to move from (x, v) to $(x+1, 1)$ with probability $\min(1, \pi(x+1)/\pi(x))$ and goes to $(x, 1)$ otherwise. Then, the new kernel is $P = qP_0 + (1-q)Q$, where P_0 is the non-reversible walk kernel introduced in Section V F. For such a kernel, there is typically no analytic expression for the stationary distribution and it is a function of both the position and the velocity. Supplementary Figure 5a and Supplementary Figure 5b display the results of a numerical experiment. For the numerical experiment, $N = 101$, $U(x) = \beta((\alpha(x-0.5))^4/4 - 1.3(\alpha(x-0.5))^2/2)$ with $\beta = 35$, $\alpha = 2.9$. The q parameter is fixed at $q = 0.9$. Supplementary Figure 5a shows that P^N and Q_N, the geometric reversibilization of P^N, have the same asymptotic rate of convergence. It also shows that their stationary measures are very close. Supplementary Figure 5b gives the targeted stationary distribution for which there is no analytic expression in the general case. In particular, it shows that, unlike the stationary measure of the equilibrium process, the distribution is velocity-dependent. The quantum algorithm based on the flat discriminant allows for efficient approximate sampling from π whereas the method transforming the singular values of the curved discriminant cannot typically be implemented because of the unknown target measure.

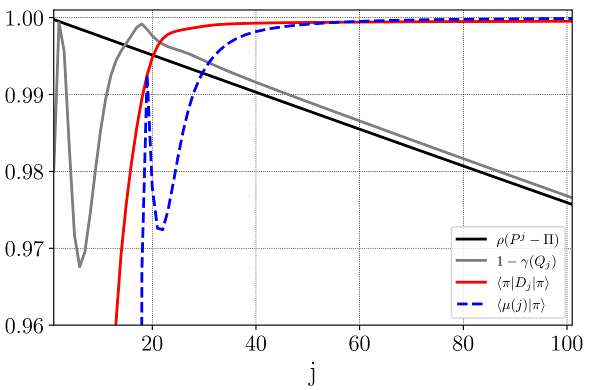

(a) Comparison between the spectral radii of P^j and its geometric reversibilization Q_j as j increases. P^N and Q_N have the same absolute spectral gap. Moreover, the leading eigenvector $|\mu(j)\rangle$ of the flat discriminant D_j has large overlap with the target state $|\pi\rangle$, as ensured by a large $\langle\pi|D_j|\pi\rangle$ parameter.

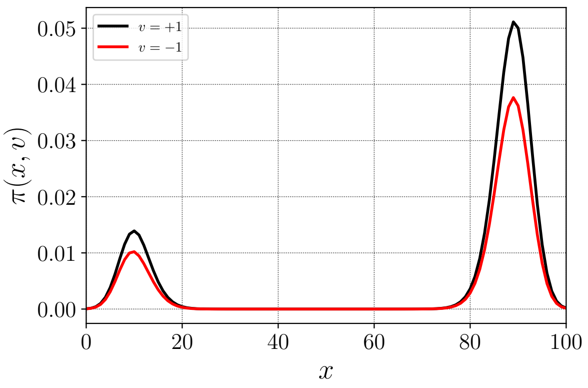

(b) Velocity-dependent stationary distribution of the out-of-equilibrium process. The distribution has, in general, no analytic expression.

Supplementary Figure 5

[1] M. Szegedy, Quantum speed-up of markov chain based algorithms, in *45th Annual IEEE Symposium on Foundations of Computer Science* (2004) pp. 32–41.
[2] A. Gilyén, Y. Su, G. H. Low, and N. Wiebe, Quantum singular value transformation and beyond: exponential improvements for quantum matrix arithmetics, in *Proceedings of the 51st Annual ACM SIGACT Symposium on Theory of Computing*, STOC '19 (ACM, 2019).
[3] S. Chatterjee, Spectral gap of nonreversible markov chains (2023), arXiv:2310.10876 [math.PR].
[4] J. M. Martyn, Z. M. Rossi, A. K. Tan, and I. L. Chuang, Grand unification of quantum algorithms, PRX Quantum **2**, 10.1103/prxquantum.2.040203 (2021).
[5] B. K. Berntson and C. Sünderhauf, Complementary polynomials in quantum signal processing, Communications in Mathematical Physics **406**, 161 (2025).
[6] C. Dolph, A current distribution for broadside arrays which optimizes the relationship between beam width and side-lobe level, Proceedings of the IRE **34**, 335 (1946).
[7] J.-W. Lee, E. Lee, Y. Lee, Y.-S. Kim, and J.-S. No, High-precision bootstrapping of RNS-CKKS homomorphic encryption using optimal minimax polynomial approximation and inverse sine function, Cryptology ePrint Archive, Paper 2020/552 (2020).
[8] D. A. Levin, Y. Peres, and E. L. Wilmer, *Markov chains and mixing times* (American Mathematical Society, 2006).

[9] D. Villemonais, *Exponential convergence to a quasi-stationary distribution and applications*, Habilitation à diriger des recherches, Université de Lorraine (Nancy) (2019).
[10] L. Miclo and P. Monmarché, Étude spectrale minutieuse de processus moins indécis que les autres, in *Séminaire de Probabilités XLV*, edited by C. Donati-Martin, A. Lejay, and A. Rouault (Springer International Publishing, Heidelberg, 2013) pp. 459–481.
[11] M. A. Nielsen and I. L. Chuang, *Quantum Computation and Quantum Information: 10th Anniversary Edition* (Cambridge University Press, 2011).
[12] B. Claudon, J. Zylberman, C. Feniou, F. Debbasch, A. Peruzzo, and J.-P. Piquemal, Polylogarithmic-depth controlled-not gates without ancilla qubits, Nature Communications **15**, 5886 (2024).
[13] J. Lemieux, B. Heim, D. Poulin, K. Svore, and M. Troyer, Efficient Quantum Walk Circuits for Metropolis-Hastings Algorithm, Quantum **4**, 287 (2020).
[14] P. Diaconis, S. Holmes, and R. M. Neal, Analysis of a nonreversible Markov chain sampler, The Annals of Applied Probability **10**, 726 (2000).
[15] C. Sünderhauf, Generalized quantum singular value transformation (2023), arXiv:2312.00723 [quant-ph].
[16] D. Motlagh and N. Wiebe, Generalized quantum signal processing, PRX Quantum **5**, 020368 (2024).

Appendix A: Quantum singular value transformations

1. Projected unitary encoding

The definitions are taken from [15].

Definition 23. Projected unitary encoding. Let A be a $N_L \times N_R$ matrix, with a so-called subnormalization $\alpha \geq \|A\|$ (the spectral norm). A projected unitary encoding $(U, \square_L, \square_R)$ consists of an $M \times M$ unitary U $(M \geq N_L, N_R)$ together with isometries $\square_L$ and $\square_R$ of dimensions $M \times N_L$ and $M \times N_R$, respectively. The encode the matrix A as

$$\square_L^\dagger U \square_R = A/\alpha. \tag{A1}$$

Definition 24. Symmetric projected unitary encoding. When A is a symmetric square matrix $(N_L = N_R = N)$, a projected unitary encoding $(U, \square_L, \square_R)$ is called symmetric projected unitary encoding when U is symmetric and $\square_L = \square_R$. In that case, one writes $(U, \square_L, \square_R) = (U, \square)$, with $\square = \square_L$.

2. Qubitization

Definition 25. Qubitized walk operator. Let $(U, \square)$ be a symmetric projected unitary encoding. Denote by $\mathcal{R}_\square$ the reflection operator about the range of $\square$, $\mathcal{R}_\square = 2\square\square^\dagger - 1$. The qubitized walk operator $\mathcal{W}$ is defined by:

$$\mathcal{W} = \mathcal{R}_\square U. \tag{A2}$$

Proposition 32. To each eigenvector-eigenvalue pair $(\lambda, |v\rangle)$ of A, there correspond eigenvector-eigenvalue pairs of $\mathcal{W}$. Define $\theta = \cos^{-1}(\lambda/\alpha) \in [0, \pi]$. If $|\lambda| < \alpha$, $\mathcal{W}$ has eigenvalues $e^{\pm i\theta}$ with eigenvectors $|\mu_\pm\rangle$ given by:

$$|\mu_\pm\rangle = \frac{1}{\sqrt{2}\sin\theta}\left(e^{\pm i\theta} - U\right)\square|v\rangle. \tag{A3}$$

If $\lambda = \pm\alpha$, $\mathcal{W}$ has eigenvalues ± 1, with corresponding eigenvector $|\mu\rangle = \square|v\rangle$ in the range of $\square$ and another one in the kernel of $\square$.

Proof. Let us start by assuming $|\lambda| < \alpha$. Compute:

$$\begin{aligned}
\mathcal{W}\left|\mu_\pm\right\rangle &= \frac{1}{\sqrt{2}\sin\theta}\mathcal{R}_\Box\left(e^{\pm i\theta}U\Box\left|v\right\rangle - U^2\Box\left|v\right\rangle\right) \\
&= \frac{1}{\sqrt{2}\sin\theta}\left(e^{\pm i\theta}2\Box\Box^\dagger U\Box\left|v\right\rangle - e^{\pm i\theta}U\Box\left|v\right\rangle - \mathcal{R}_\Box\Box\left|v\right\rangle\right), \text{because } U \text{ is symmetric and therefore } U^2 = 1 \\
&= \frac{1}{\sqrt{2}\sin\theta}\left(e^{\pm i\theta}2\Box(\lambda/\alpha)\left|v\right\rangle - e^{\pm i\theta}U\Box\left|v\right\rangle - \Box\left|v\right\rangle\right) \\
&= \frac{1}{\sqrt{2}\sin\theta}(e^{\pm i\theta}(e^{\pm i\theta} + e^{\mp i\theta}) - e^{\pm i\theta}U - 1)\Box\left|v\right\rangle \\
&= \frac{e^{\pm i\theta}}{\sqrt{2}\sin\theta}\left(e^{\pm i\theta} + e^{\mp i\theta} - e^{\mp i\theta} - U\right)\Box\left|v\right\rangle \\
&= \frac{e^{\pm i\theta}}{\sqrt{2}\sin\theta}(e^{\pm i\theta} - U)\Box\left|v\right\rangle \\
&= e^{\pm i\theta}\left|\mu_\pm\right\rangle.
\end{aligned} \tag{A4}$$

If $\lambda = \pm\alpha$, then $\theta = 0, \pi$ and:

$$\mathcal{R}_\Box U\left|v\right\rangle = \pm 2\Box\left|v\right\rangle - U\Box\left|v\right\rangle. \tag{A5}$$

However, the norms of $\mathcal{R}_\Box U\left|v\right\rangle$, $\Box\left|v\right\rangle$ and $U\Box\left|v\right\rangle$ are 1, so $\mathcal{R}_\Box U\left|v\right\rangle = \pm\Box\left|v\right\rangle$. □

3. Hermitianization

Definition 26. Hermitianization. Let A be $N_L \times N_R$ complex matrix, with corresponding projected unitary encoding $(U, \Box_L, \Box_R)$. It is possible to construct a symmetric projected unitary encoding of the $\overline{N}\times\overline{N}$ ($\overline{N} = N_L + N_R$) matrix

$$\overline{A}/\alpha = \begin{pmatrix} 0 & A/\alpha \\ A^\dagger/\alpha & 0 \end{pmatrix}. \tag{A6}$$

The symmetric projected unitary $(\overline{U}, \overline{\Box})$ has double the dimension of U and is defined by:

$$\overline{U} = \left(\left|0\right\rangle\left\langle 0\right| \otimes U + \left|1\right\rangle\left\langle 1\right| \otimes U^\dagger\right)(X \otimes 1) \tag{A7}$$

and

$$\overline{\Box} = (\left|0\right\rangle\left\langle 0\right| \otimes \Box_L + \left|1\right\rangle\left\langle 1\right| \otimes \Box_R). \tag{A8}$$

The qubitized walk operator of $\overline{U}$ can readily be constructed thanks to the reflection $\mathcal{R}_{\overline{\Box}} = \left|0\right\rangle\left\langle 0\right| \otimes \mathcal{R}_{\Box_L} + \left|1\right\rangle\left\langle 1\right| \otimes \mathcal{R}_{\Box_R}$.

4. Generalized quantum signal processing

This section comes from [16]. Let U be a unitary. Define the 0-controlled U operation $A = \left|0\right\rangle\left\langle 0\right| \otimes U + \left|1\right\rangle\left\langle 1\right| \otimes 1$. Also, let us use the following real parametrization of $\mathsf{SU}(2)$ matrices:

$$R(\theta, \phi, \lambda) = \begin{pmatrix} e^{i(\lambda+\phi)}\cos\theta & e^{i\phi}\sin\theta \\ e^{i\lambda}\sin\theta & -\cos\theta \end{pmatrix}. \tag{A9}$$

Matrices denotes by $R(\theta, \phi, \lambda)$ will always apply to the ancilla (the one that controls the U gate).

Theorem 3. Generalized quantum signal processing. Fix $d \in \mathbb{N}$. Then, the two following statements are equivalent.

1. There exists $\theta, \phi \in \mathbb{R}^{d+1}$, $\lambda \in \mathbb{R}$ such that:

$$\left(\prod_{j=1}^{d} R(\theta_j, \phi_j, 0) A\right) R(\theta_0, \phi_0, \lambda) = \begin{pmatrix} P(U) & \cdot \\ Q(U) & \cdot \end{pmatrix}. \tag{A10}$$

2. P, Q are complex polynomials of degree at most d and such that $|P|^2 + |Q|^2 = 1$ on the complex unit circle.

Theorem 4. For each complex polynomial P, the following two statements are equivalent.

1. $|P|^2 \leq 1$ on the complex unit circle.
2. There exists a complex polynomial Q of degree equal to that of P and such that on the complex unit circle: $|P|^2 + |Q|^2 = 1$.

Corollary 8. For each complex polynomial P, the following two statements are equivalent.

1. $|P|^2 \leq 1$ on the complex unit circle.
2. There exists $\theta, \phi \in \mathbb{R}^{d+1}$, $\lambda \in \mathbb{R}$ such that:

$$\left(\prod_{j=1}^{d} R(\theta_j, \phi_j, 0) A\right) R(\theta_0, \phi_0, \lambda) = \begin{pmatrix} P(U) & \cdot \\ \cdot & \cdot \end{pmatrix}. \tag{A11}$$

Moreover, it is possible to compute Q for P efficiently up to $\deg(P) \approx 10^7$. Given P, Q of degree d, the phases $\theta, \phi \in \mathbb{R}^{d+1}$, $\lambda \in \mathbb{R}$ can be computed in time $\tilde{\mathcal{O}}(d)$. An explicit representation of a valid complementary polynomial may be found in [5]. A valid complementary polynomial is given by:

$$\forall z \in \partial B_1(0) : Q(z) = (z^2 - 1) \exp\left(\frac{1}{2} a_0 + \sum_{n=1}^{\infty} a_n z^n\right), \tag{A12}$$

where $\log\left(\frac{1-|P(e^{i\theta})|^2}{|e^{2i\theta}-1|^2}\right) = \sum_{n\in\mathbb{Z}} a_n e^{in\theta}$ for all $\theta \in \mathbb{R}$.

5. Generalized quantum eigenvalue transformation

Theorem 5. Generalized quantum eigenvalue transformation Let $(U, \square)$ be a symmetric projected encoding of the symmetric matrix A/α. Consider a degree-d complex polynomial

$$\upsilon(x) = \sum_{n=0}^{d} a_n T_n(x), \tag{A13}$$

written in the basis of Chebyshev polynomials. Consider its associated signal processing polynomial $\Upsilon(z) = \sum_{n=0}^{d} a_n z^n$. Assume that $\max_{z\in\partial B_1(0)} |\Upsilon(z)| \leq 1$. Let $\mathcal{G}$ be the generalized quantum signal processing of $\mathcal{R}_\square U$ applying the polynomial Υ. Then, $(\mathcal{G}, |0\rangle \otimes \square)$ is a symmetric projected encoding of $\upsilon(A/\alpha)$.

Proof. Let $|v_i\rangle, |v_j\rangle$ be eigenvectors of A/α with eigenvalues λ_i, λ_j. According to proposition 32, $\square |v_i\rangle = \frac{|\mu_i^+\rangle - |\mu_i^-\rangle}{i\sqrt{2}}$ and $\square |v_j\rangle = \frac{|\mu_j^+\rangle - |\mu_j^-\rangle}{i\sqrt{2}}$. Therefore,

$$\begin{aligned}
\langle v_j| \left(\langle 0| \otimes \square^\dagger \mathcal{G} |0\rangle \otimes \square\right) |v_i\rangle &= \frac{1}{i\sqrt{2}} \langle v_j| \square^\dagger \left(\langle 0| \otimes 1\mathcal{G} |0\rangle \otimes 1\right) \left(|\mu_i^+\rangle - |\mu_i^-\rangle\right) \\
&= \frac{1}{i\sqrt{2}} \langle v_j| \left[\left(\Upsilon(e^{i\theta_j}) |\mu_i^+\rangle - \Upsilon(e^{-i\theta_j}) |\mu_i^-\rangle\right)\right] \\
&= \frac{1}{2} \left(\langle \mu_j^+| - \langle \mu_j^-|\right) \left[\left(\Upsilon(e^{i\theta_j}) |\mu_i^+\rangle - \Upsilon(e^{-i\theta_j}) |\mu_i^-\rangle\right)\right] \\
&= \frac{\Upsilon(e^{i\theta_j}) + \Upsilon(e^{-i\theta_j})}{2} \delta_{ij}.
\end{aligned} \tag{A14}$$

Conclude by using the Chebyshev polynomial identity $T_k(x) = \frac{e^{ikx}+e^{-ikx}}{2}$ valid for all $k \in \mathbb{N}, \theta \in \mathbb{R}$:

$$\langle v_j| \left(\langle 0| \otimes \square^\dagger \mathcal{G} |0\rangle \otimes \square\right) |v_i\rangle = \upsilon(\lambda_j/\alpha)\delta_{ij}. \tag{A15}$$

□

Definition 27. Scaling factor. Let υ be a complex polynomial of degree d written in the Chebyshev basis: $\upsilon(x) = \sum_{n=0}^{d} a_n T_n(x)$. Define the corresponding polynomial $\Upsilon(z) = \sum_{n=0}^{d} a_n z^j$ with the same coefficients when expressed in the monomial basis. For such υ, define the scaling down factor β by:

$$\beta = \frac{\max_{z\in\partial B_1(0)} |\Upsilon(z)|}{\max_{x\in[-1,1]} |\upsilon(x)|}. \tag{A16}$$

Note that the scaling factor is defined to ensure that $\upsilon(x)/\beta$ can be applied to the eigenvalues of a symmetric operator encoded in a projected symmetric unitary encoding. The success probability of the block-encoding is multiplied, scaled-down, by $1/\beta^2$.

6. Generalized quantum singular value transformation

Definition 28. Singular value transformation by defined-parity polynomials. Let A be an arbitrary complex $N_L \times N_R$ matrix. It admits singular value decomposition $A = W^\dagger D V$, with $A|v_i\rangle = \sigma_i |w_i\rangle$. Here, V is a $N_L \times N_L$ unitary matrix, W a $N_R \times N_R$ unitary and D a $N_L \times N_R$ with the $\min(N_L, N_R)$ singular values $(\sigma_i)_{i=0}^{\min(N_L,N_R)-1}$ arranged on the diagonal. The singular value transformation by even $p_e(x)$ or odd $p_o(x)$ polynomials are defined by:

$$\begin{cases} p_o(A/\alpha) = W^\dagger p_o(D/\alpha) V \\ p_e(A/\alpha) = V^\dagger p_e(D'/\alpha) V, \end{cases} \tag{A17}$$

with D' being D restricted to a $N_R \times N_R$ diagonal which keeps all the singular values $(\sigma_i)_{i=0}^{\min(N_L,N_R)-1}$.

Proposition 33. Let A be an arbitrary complex $N_L \times N_R$ matrix. Denote the right singular vectors of A/α by $(|v_i\rangle)$ and its left singular vectors by $(|w_i\rangle)$. The eigen-decomposition of the hermitianization $\overline{A}/\alpha$ has eigenvector-eigenvalue decomposition given by:

$$\frac{1}{\alpha}\overline{A}\frac{1}{\sqrt{2}}\begin{pmatrix} |w_i\rangle \\ \pm|v_i\rangle \end{pmatrix} = \pm\sigma_i \frac{1}{\sqrt{2}}\begin{pmatrix} |w_i\rangle \\ \pm|v_i\rangle \end{pmatrix}. \tag{A18}$$

Proof. This is a straightforward computation:

$$\frac{1}{\alpha}\overline{A}\frac{1}{\sqrt{2}}\begin{pmatrix} |w_i\rangle \\ \pm|v_i\rangle \end{pmatrix} = \frac{1}{\alpha}\begin{pmatrix} 0 & A \\ A^\dagger & 0 \end{pmatrix}\frac{1}{\sqrt{2}}\begin{pmatrix} |w_i\rangle \\ \pm|v_i\rangle \end{pmatrix} = \pm\sigma_i \frac{1}{\sqrt{2}}\begin{pmatrix} |w_i\rangle \\ \pm|v_i\rangle \end{pmatrix}. \tag{A19}$$

□

Proposition 34. Consider a polynomial $p(x)$ having for even part $p_e(x)$ and odd part $p_o(x)$. Let $\mathfrak{G}$ be the generalized quantum eigenvalue transform applying $p(x)$ to the hermitianized projected unitary encoding $\overline{U}$ of $\overline{A}/\alpha$. Then,

$$\langle 0| \otimes \overline{\square}^\dagger \mathfrak{G} |0\rangle \otimes \overline{\square} = \begin{pmatrix} W^\dagger p_e(D/\alpha) W & p_o(A/\alpha) \\ (p_o(A/\alpha))^\dagger & p_e(A/\alpha) \end{pmatrix}. \tag{A20}$$

Proof. Let us prove the proposition by considering the action of $\langle 0| \otimes \overline{\square}^\dagger \mathfrak{G} |0\rangle \otimes \overline{\square}$ on basis vectors of the form:

$$\begin{pmatrix} |w_i\rangle \\ 0 \end{pmatrix}, \begin{pmatrix} 0 \\ |v_i\rangle \end{pmatrix}. \tag{A21}$$

Compute:

$$\begin{aligned} \langle 0| \otimes \overline{\square}^\dagger \mathfrak{G} |0\rangle \otimes \overline{\square} \begin{pmatrix} |w_i\rangle \\ 0 \end{pmatrix} &= \frac{1}{2} \langle 0| \otimes \overline{\square}^\dagger \mathfrak{G} |0\rangle \otimes \overline{\square} \left[\begin{pmatrix} |w_i\rangle \\ |v_i\rangle \end{pmatrix} + \begin{pmatrix} |w_i\rangle \\ -|v_i\rangle \end{pmatrix}\right] \\ &= \frac{1}{2}\left[p(\sigma_i)\begin{pmatrix} |w_i\rangle \\ |v_i\rangle \end{pmatrix} + p(-\sigma_i)\begin{pmatrix} |w_i\rangle \\ -|v_i\rangle \end{pmatrix}\right] \\ &= \begin{pmatrix} p_e(\sigma_i)|w_i\rangle \\ p_e(\sigma_i)|v_i\rangle \end{pmatrix}. \end{aligned} \tag{A22}$$

One now recognizes the first column of the right-hand side of equation A20. Regarding the second column, compute:

$$\begin{aligned}
\langle 0| \otimes \overline{\square}^{\dagger} \mathfrak{G} \, |0\rangle \otimes \overline{\square} \begin{pmatrix} 0 \\ |v_i\rangle \end{pmatrix} &= \frac{1}{2} \langle 0| \otimes \overline{\square}^{\dagger} \mathfrak{G} \, |0\rangle \otimes \overline{\square} \left[\begin{pmatrix} |w_i\rangle \\ |v_i\rangle \end{pmatrix} - \begin{pmatrix} |w_i\rangle \\ -|v_i\rangle \end{pmatrix} \right] \\
&= \frac{1}{2} \left[p(\sigma_i) \begin{pmatrix} |w_i\rangle \\ |v_i\rangle \end{pmatrix} - p(-\sigma_i) \begin{pmatrix} |w_i\rangle \\ -|v_i\rangle \end{pmatrix} \right] \\
&= \begin{pmatrix} p_o(\sigma_i) \, |w_i\rangle \\ p_e(\sigma_i) \, |v_i\rangle \end{pmatrix}.
\end{aligned} \tag{A23}$$

□

Appendix B: Amplitude amplification

The amplitude amplification algorithm described in the quantum singular value framework can be found in [4]. Assume that we are given some unitary $U = 2\,|\pi\rangle\langle\pi| - 1$ for some state $|\pi\rangle$ and that we know how to prepare some state $|\alpha\rangle$ such that $a = |\langle\alpha|\pi\rangle| \neq 0$. The goal is to construct a unitary V such that:

$$\| \, |\pi\rangle\langle\pi| \, V \, |\alpha\rangle\langle\alpha| - |\pi\rangle\langle\alpha| \, \| < \epsilon, \tag{B1}$$

for some precision $0 < \epsilon < 1$. From Proposition 6, there exists an odd polynomial of degree $\mathcal{O}\left(\frac{\log(1/\epsilon)}{a}\right)$ that can be applied to the 1×1 matrix a such that Equation B1 is verified.

Appendix C: Distances between probability measures

Proposition 35. For any probability distributions p, q on $\mathbb{S}$:

$$1 - \langle p|q\rangle \leq d_{TV}(p,q) \leq \sqrt{1 - \langle p|q\rangle^2}. \tag{C1}$$

Proof. Using the triangle inequality:

$$\begin{aligned}
1 - \langle q|p\rangle &= \frac{1}{2} \sum_x (\sqrt{p(x)} - \sqrt{q(x)})^2 \\
&\leq \frac{1}{2} \sum_x |\sqrt{p(x)} - \sqrt{q(x)}||\sqrt{p(x)} + \sqrt{q(x)}| \\
&= \frac{1}{2} \sum_x |p(x) - q(x)|.
\end{aligned} \tag{C2}$$

By Cauchy-Schwarz:

$$\begin{aligned}
\frac{1}{2} \sum_x |p(x) - q(x)| &= \frac{1}{2} \sum_x |\sqrt{p(x)} - \sqrt{q(x)}||\sqrt{p(x)} + \sqrt{q(x)}| \\
&\leq \frac{1}{2} \sqrt{(2 - 2\,\langle p|q\rangle)(2 + 2\,\langle p|q\rangle)} \\
&= \sqrt{1 - \langle p|q\rangle^2}.
\end{aligned} \tag{C3}$$

□

Appendix D: Perron-Frobenius Theorem

Theorem 6. Let A be an $n \times n$ irreducible matrix with positive coefficients. Then the spectral radius of A is a simple eigenvalue of A and the corresponding eigenspace is generated by a vector with strictly positive components.

Appendix E: Bauer-Fike Theorem

Theorem 7. Bauer-Fike Theorem. Let A be a diagonalizable complex $n \times n$ matrix, $A = PDP^{-1}$ with P an invertible matrix and $D = \text{diag}(\lambda_1, ..., \lambda_n)$. Let H be any matrix. Then,

$$\sigma(A+H) \subset \bigcup_{\lambda \in \sigma(A)} B_\epsilon(\lambda), \tag{E1}$$

where $\epsilon = \|P\| \|P^{-1}\| \|H\|$. Moreover, if $I \subset \{1, ..., n\}$ is such that,

$$\cup_{i \in I} B_\epsilon(\lambda_i) \cap \cup_{i \notin I} B_\epsilon(\lambda_i) = \emptyset, \tag{E2}$$

then the number of eigenvalues of $A + H$ in $\cup_{i \in I} B_\epsilon(\lambda_i)$ is exactly $|I|$.

Note that if A is normal (for example symmetric), then P can be chosen to be unitary such that $\|P\| = \|P^{-1}\| = 1$.

Appendix F: Doeblin condition

The Doeblin condition, introduced below, is useful to bound mixing times. It is also useful to show that the mixing time does not depend on certain parameters.

Definition 29. A Markov kernel P on $\mathbb{S}$ is said to satisfy the Doeblin condition with constant $0 < \alpha \leq 1$ if:

$$\forall x, y \in \mathbb{S} : d_{TV}(P(x, \cdot), P(y, \cdot)) \leq 1 - \alpha. \tag{F1}$$

Proposition 36. Assume that there exists $C > 0$ such that $P^n(x, y) \geq \frac{C}{n}$ for each $x, y \in \mathbb{S}$ and n large enough. Then,

$$\exists \alpha > 0, n_0 \in \mathbb{N} : \forall x, y \in \mathbb{S}, n \geq n_0 : d_{TV}(P^n(x, \cdot), P^n(y, \cdot)) \leq 1 - \alpha. \tag{F2}$$

In other words, P^n satisfies a Doeblin condition with constant independent from the state space size n.

Proof. Keep in mind the fact that the lines of P must sum to 1 and therefore $C \leq 1$. Then, note that for each $x, y \in \mathbb{S}$:

$$\langle P^n(x, \cdot) | P^n(y, \cdot) \rangle \geq \sum_z \frac{C}{n} = C. \tag{F3}$$

Proposition 35 then implies $d_{TV}(P^n(x, \cdot), P^n(y, \cdot)) \leq \sqrt{1 - C^2}$. Letting $\alpha = 1 - \sqrt{1 - C^2} \in]0, 1]$ finishes the proof. □
